# Tailored Electro–Magnetic–Porous Multigradient Nanoarchitectonics for Absorption‐Dominated Electromagnetic Interference Shielding and Adaptive Multifunctionality

**DOI:** 10.1002/advs.202511234

**Published:** 2025-08-14

**Authors:** Runze Shao, Guilong Wang, Wenyu Wang, Jialong Chai, Guoqun Zhao, Guizhen Wang

**Affiliations:** ^1^ State key Laboratory of Advanced Equipment and Technology for Metal Forming Shandong University Jinan Shandong 250061 China; ^2^ Key Laboratory for Liquid‐Solid Structural Evolution and Processing of Materials (Ministry of Education) Shandong University Jinan Shandong 250061 China; ^3^ School of Control Science and Engineering Shandong University Jinan Shandong 250061 China; ^4^ Center for Advanced Studies in Precision Instruments Hainan University Haikou Hainan 570228 China

**Keywords:** dual‐nanofibrous structure, electro–magnetic–porous multigradient, emi shielding, flexible wearable, multifunctional

## Abstract

Herein, a multifunctional electromagnetic interference (EMI) shielding membrane with a conductivity–permeability–pore size gradient structure is fabricated via shear‐induced in situ fibrillation coupled with layer‐by‐layer assembly. Under intense shear, carbon nanotube (CNT) nanofibers intertwine with polytetrafluoroethylene (PTFE) nanofibrils to form an interpenetrating dual‐nanofibrous network, which not only robustly anchors Fe_3_O_4_ nanoparticles but also establishing continuous conductive and thermal pathways. Moreover, the integration of this unique dual‐nanofibrous structure with the intrinsic properties of its components endows the PTFE/CNT/Fe_3_O_4_‐gradient (FCFe‐G) membrane with superior mechanical properties, superhydrophobicity, flame retardancy, and corrosion resistance. More importantly, leveraging a multigradient‐induced “impedance matching–multilevel polarization–reabsorption” synergistic mechanism, the FCFe‐G membrane (101.1 µm) achieves absorption‐dominated EMI shielding with an exceptional EMI shielding effectiveness (SE) of 53.79 dB and ultralow reflectivity (0.38). Furthermore, anisotropic thermal management and CNT‐driven negative temperature coefficient behavior facilitate rapid heat dissipation and early fire warning. The film's dual‐mode electro/photothermal response further enables aerospace deicing, medical hyperthermia, and antibacterial applications. This work introduces a “composition–structure multigradient” design paradigm, offering a promising strategy for intelligent EMI shielding in aerospace, flexible electronics, and smart wearables.

## Graphical abstract

An electro–magnetic–porous multigradient nanofibrous electromagnetic interference (EMI) membrane is fabricated via shear‐induced in situ fibrillation and layer‐by‐layer assembly. Leveraging a multigradient‐induced “impedance matching–polarization–reabsorption” synergistic mechanism, the membrane achieves absorption‐dominated high‐efficiency EMI shielding performance while demonstrating superior mechanical performance, superhydrophobicity, fire warning, and extraordinary electro/photothermal conversion capabilities.

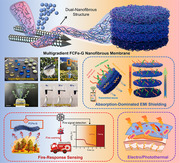

## 1 Introduction

The deep integration of fifth‐generation (5G) mobile communication and Internet of Things (IoT) technologies has driven the explosive growth of portable and wearable electronic devices.^[^ ^1^, ^2^, ^3^ ^]^ However, the dense deployment of such devices inevitably generates electromagnetic interference (EMI) and radiation, which not only compromise the stable operation of precision electronic systems through near‐field coupling effects, but also pose substantial risks to human health.^[^ ^4^, ^5^, ^6^ ^]^ Although conventional metallic shielding materials exhibit excellent EMI shielding effectiveness (SE), their inherent drawbacks—including high density, susceptibility to corrosion, and poor processability—severely limit their application in flexible electronics.^[^ ^7^, ^8^ ^]^ More critically, the pronounced impedance mismatch at metal interfaces results in strong reflection of electromagnetic waves (EMWs), inducing secondary radiation and exacerbating signal crosstalk among integrated devices, thereby creating a vicious cycle.

In this context, porous conductive polymer composites (CPCs) constructed from lightweight polymer matrices have established a new paradigm for electromagnetic (EM) protection, owing to their low density, high flexibility, corrosion resistance, and structural tunability.^[^ ^9^, ^10^, ^11^ ^]^ By synergistically regulating percolating conductive networks and hierarchical porous architectures, CPCs enable efficient dissipation of EMWs through simultaneous absorption and multiple scattering/reflection mechanisms.^[^ ^12^, ^13^ ^]^ To date, a variety of CPCs have been developed for high‐performance EMI shielding, including carbon‐based composites, graphene‐assembled structures, and MXene‐reinforced composite aerogels.^[^ ^14^, ^15^, ^16^ ^]^ Nevertheless, current strategies for enhancing the shielding capabilities of CPCs are still constrained by intrinsic trade‐offs. Increasing the concentration of conductive fillers, while effective in boosting electrical conductivity and EMI shielding efficiency, inevitably deteriorates filler–polymer interfacial compatibility and exacerbates EMW reflection.^[^ ^17^ ^]^ On the other hand, thickness‐dependent approaches to improving shielding directly conflict with the imperative for ultrathin, lightweight designs demanded by flexible electronic devices.^[^ ^18^ ^]^ Therefore, achieving cross‐dimensional synergy among conductive network topology, hierarchical pore distribution, and EM parameter modulation within submillimeter thicknesses has emerged as a crucial breakthrough toward resolving the inherent trade‐off between high shielding performance and low EM reflection.

Resolving this paradox hinges on achieving precise synergy between EM parameters and structural features through spatially heterogeneous design, thereby enabling the integration of both anti‐reflection and high‐efficiency shielding functionalities within a single asymmetric architecture.^[^ ^19^ ^]^ Traditional strategies, such as Janus structures or alternating multilayer configurations, can mitigate EM reflection via reflection–reabsorption cycling mechanisms.^[^ ^20^, ^21^, ^22^ ^]^ However, their performance is fundamentally constrained by abrupt conductivity transitions that induce interfacial impedance mismatches, as well as weak interlayer coupling caused by complex fabrication processes.^[^ ^23^ ^]^ In contrast, conductivity‐gradient structures based on impedance matching theory offer a more promising solution.^[^ ^24^ ^]^ Gradual conductivity variations from surface to interior facilitate continuous impedance modulation, ensuring effective air–material interface matching.^[^ ^25^ ^]^ Nonetheless, according to Schelkunoff's transmission theory, dielectric loss alone is insufficient to suppress reflection effectively.^[^ ^15^ ^]^ Introducing inverse magnetic permeability gradients can synergistically activate magnetic hysteresis loss, eddy current dissipation, and natural resonance effects, thereby constructing a multi‐physics‐coupled EMW dissipation network.^[^ ^26^ ^]^

Building upon these gradient‐based strategies, integrating hierarchical pore size gradient introduces a paradigm shift in EMW manipulation by leveraging the structural dimension as an independent design variable. On one hand, continuous pore size transitions from the material surface to the bulk enable stepwise impedance matching as EMW penetrate the material, thereby minimizing reflection at each interface.^[^ ^27^ ^]^ On the other hand, interconnected hierarchical microporous structure not only enhance the EMW penetration depth but also amplify interfacial polarization and prolong EMW propagation paths through multiple reflections and scattering.^[^ ^28^, ^29^, ^30^ ^]^ Despite these theoretical advantages, existing fabrication methods struggle to decouple electrical, magnetic, and pore size gradients and to implement them simultaneously without sacrificing structural integrity. Consequently, most studies have focused exclusively on compositional (electrical or magnetic) gradients, while the role of structural gradients as active parameters for modulating EMW propagation and attenuation remains underexplored. Hence, developing a facile yet robust strategy to fabricate durable, multifunctional CPCs with integrated electro–magnetic–porous multigradient structure—capable of synergistic impedance matching, energy dissipation, and interfacial modulation across multiple physical dimensions—remains a critical technological bottleneck demanding urgent requires resolution.

In this study, we successfully fabricated PTFE/CNT/Fe_3_O_4_‐Gradient (FCFe‐G) composites with a triple‐gradient structure of electrical conductivity, magnetic permeability, and pore size by integrating shear‐induced in situ fibrillation with a layer‐by‐layer (LBL) assembly strategy. This approach first utilizes a high‐shear field to induce PTFE fibrillation, which interweaves with carbon nanotube (CNT) fibers to form an interlocked dual‐nanofibrous network.^[^ ^31^ ^]^ This unique structure not only effectively immobilizes Fe_3_O_4_ nanoparticles, but also features an internal porous structure that promotes the synergistic enhancement of magnetic and dielectric losses through multiple reflections and scattering.^[^ ^32^ ^]^ Building upon this, an Opera cake–inspired layered assembly process was employed to achieve a continuous gradient transition from a highly conductive surface layer to a high magnetic loss inner layer within a thickness of 100 µm. Simultaneously, a hierarchical pore gradient structure was created to optimize impedance matching characteristics. Finite element simulations and experimental evaluations confirm that the multimodal gradient design of FCFe‐G enables a synergistic “impedance matching–multi‐level polarization–reabsorption” mechanism, achieving a high EMI SE of 53.79 dB and an absorption coefficient (A) of 0.62 in the X‐band. Impressively, the normalized specific SE (SSE) reaches 9539.52 dB·cm^2^·g^−1^, demonstrating the integration of robust shielding and efficient anti‐reflection capabilities. Moreover, the structural–compositional synergy endows the FCFe‐G membrane with multifunctionality, including outstanding mechanical flexibility, superhydrophobicity, efficient heat dissipation, fire‐response sensing, and electro/photothermal conversion. This work presents a universal and rational design methodology for multimodal gradient materials, propelling the advancement of lightweight EM functional materials toward cross‐dimensional synergy and multi‐scenario adaptability.

## 2 Result and Discussion

### 2.1 Fabrication and Characterization of the FCFe‐G Multigradient Membrane

**Figure 1:**
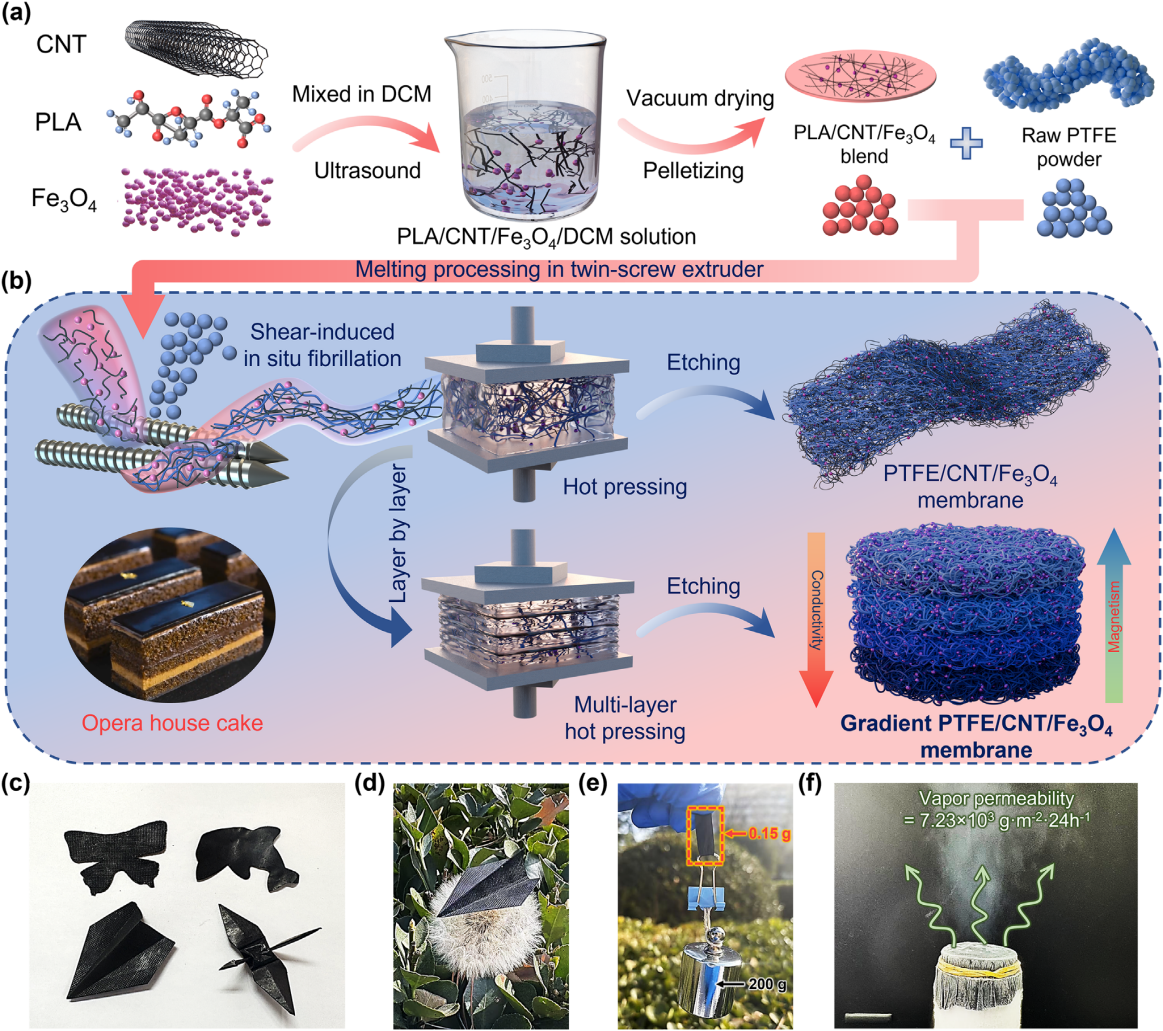
Preparation of the FCFe‐G multigradient nanofibrous membrane. a) Schematic illustration of the solution blending process. b) Schematic of the shear‐induced in situ fibrillation and LBL stacking process. c) Demonstration of the ultraflexibility of the FCFe‐G membrane, which can be cut and folded into complex shapes. d) Photograph showing a 0.15 g FCFe‐G membrane supporting a 200 g weight without noticeable deformation. e) A paper airplane folded from the FCFe‐G membrane resting on a dandelion seed. f) Photograph of liquid nitrogen vapor permeating through the FCFe‐G membrane.

The FCFe‐G composite membrane was fabricated via a synergistic strategy combining shear‐induced in situ fibrillation and LBL assembly (**Figure** **1**a,b). To ensure uniform dispersion of EM functional fillers within the PTFE nanofibrous network, CNTs and Fe_3_O_4_ nanoparticles were first blended with polylactic acid (PLA) in dichloromethane (DCM). The resulting CNT/Fe_3_O_4_/PLA mixture was vacuum‐dried, pelletized, and then melt‐compounded with PTFE powder in a twin‐screw extruder. During this process, PLA served as a lubricant to transfer shear stress generated by the screws to PTFE particles, while the unique surface characteristics and loosely packed intercrystalline structure of PTFE crystals facilitated disentanglement and in situ fibrillation under intense shear fields.^[^ ^33^, ^34^ ^]^ This generated an intertwined dual‐nanofibrous network of PTFE fibrils and CNT nanofibers, which robustly immobilized Fe_3_O_4_ nanoparticles. Inspired by the multilayer architecture of the French dessert “Opera Cake,” precursor films with graded CNT contents (10 to 40 wt.%) were sequentially stacked and hot‐pressed to form a continuous gradient structure. Finally, selective removal of PLA by DCM etching yielded a hierarchical FCFe‐G nanofibrous membrane featuring integrated gradients in electrical conductivity, magnetic permeability, and pore size.

As shown in Figure 1c and Figure S1a, Supporting Information, the FCFe‐G membrane exhibits a matte black appearance with surface‐aligned textural patterns, which demonstrates exceptional mechanical compliance that enables precise cutting or folding into complex geometries. The intrinsic mechanical robustness imparted by its unique dual‐nanofibrous network endows the FCFe‐G membrane (0.558 g·cm^−3^) featuring ultralight yet durable characteristics (Table S1, Supporting Information). As illustrated in Figure 1d,e, the membrane can be effortlessly supported by a dandelion seed while bearing a load over 1300 times its own weight without noticeable deformation. Additionally, the hierarchically porous structure further confers exceptional breathability, enabling unimpeded liquid nitrogen permeation through the FCFe‐G membrane even under sealed conditions (Figure 1f). Therefore, the wearability of the FCFe‐G membrane was further tested. The FCFe‐G membrane exhibits a water vapor transmission rate (WVTR) of 7.23×10^3^ g·m^−2^·24h^−1^, rivaling or exceeding common breathable textiles such as cotton and polyester (Figure S1b, Supporting Information). This result indicates that the nanopores in the FCFe‐G membrane are sufficiently permeable to transmit water vapor from perspiration by natural diffusion and convection. This characteristic not only effectively prevents the accumulation of water vapor and improves overall comfort, but also helps to balance the internal and external pressures of the equipment.^[^ ^35^ ^]^ Moreover, the embedded CNT network provides outstanding ultraviolet (UV) shielding. As depicted in Figure S1c, Supporting Information, the FCFe‐G membrane exhibits near‐zero UV transmittance (<0.2%) across the 200–400 nm range, achieving an ultraviolet protection factor (UPF) of 9785—approximately three orders of magnitude higher than that of cotton (15.8), polyamide (24.8), or polyester (19.2). The synergistic integration of these properties renders the FCFe‐G membrane an ideal candidate for durable, skin‐adaptive, and breathable wearable electronics.

**Figure 2:**
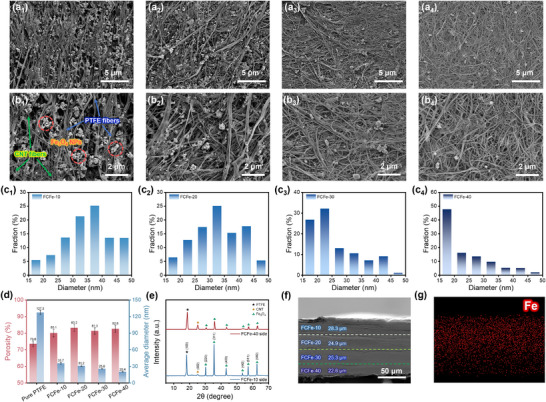
Microstructural morphology and structural characterization of FCFe and FCFe‐G membranes. Surface morphologies of a_1_, b_1_) FCFe‐10, a_2_, b_2_) FCFe‐20, a_3_, b_3_) FCFe‐30, and a_4_, b_4_) FCFe‐40 membranes. Pore size distribution curves of c_1_) FCFe‐10, c_2_) FCFe‐20, c_3_) FCFe‐30, and c_4_) FCFe‐40. d) Trends in porosity and average pore size of single‐layer FCFe membranes. e) XRD patterns of both sides of the FCFe‐G membrane. f) Cross‐sectional morphology of the FCFe‐G membrane. g) Cross‐sectional elemental mapping of the FCFe‐G membrane.

The microstructure of FCFe single‐layer membranes is shown in **Figure** **2**a. Its internal framework consists of two distinct nanofiber populations: the thicker and straighter fibers are identified as PTFE nanofibrils, whereas the thinner, more sinuous, and randomly oriented fibers correspond to CNT nanofibers. Statistical analysis reveals a bimodal diameter distribution, with PTFE fibrils centered at 100–200 nm and CNT fibers concentrated below 50 nm (Figure S2, Supporting Information). Driven by strong shear fields, these mechanically complementary nanofibers interlock to form a dual‐nanofibrous network that efficiently immobilizes Fe_3_O_4_ nanoparticles while facilitates the formation of a highly porous structure (Figure 2b). As shown in Figure 2c,d, the FCFe membrane exhibits a unique pore evolution mechanism: as the CNT content increases from 10 to 40 wt.%, the overall porosity remains stable at 81.8% (8.2% higher than that of pure PTFE), yet the average pore diameter sharply decreases from 35.7 nm to 20.4 nm. This decoupled modulation of porosity and pore size arises from competing topological effects between bimodal fibers. With increasing CNT content, the high–aspect‐ratio CNT networks act as “nanoscale blades,” slicing PTFE‐derived macropores into densely packed small pores.^[^ ^31^ ^]^ Concurrently, Fe_3_O_4_ nanoparticles interspersed among the nanofibers progressively reinforce the network structure, facilitating the generation of additional pores. Both CNT and Fe_3_O_4_ jointly contribute to pore formation, thereby stabilizing the overall porosity despite structural refinement.^[^ ^32^ ^]^ Accordingly, a hierarchical gradient FCFe‐G membrane was constructed by LBL stacking four porous FCFe nanofibrous layers with ascending CNT contents. This yielded a continuous increase in electrical conductivity, a corresponding decrease in magnetic permeability, and a systematic refinement of the pore structure.

The wide‐angle X‐ray diffraction (WAXD) patterns reveal that the incorporation of CNT and Fe_3_O_4_ exerts minimal influence on the original crystalline structure of PTFE, indicating that the composite components remain largely independent. In this system, CNT and PTFE assemble into a dual‐nanofibrous network via physical entanglement rather than chemical interaction (Figure 2e).^[^ ^36^ ^]^ Figure 2f shows the cross‐sectional scanning electron microscope (SEM) image of the FCFe‐G membrane, demonstrating a well‐integrated and stacked layered structure without visible delamination between adjacent layers. This tight interfacial bonding is attributed to the PLA‐template‐assisted LBL hot‐pressing process, in which van der Waals interactions and fiber pinning effects promote robust mechanical interlocking at the multilayer interfaces. Furthermore, energy‐dispersive X‐ray spectroscopy (EDS) analysis of the cross‐section reveals a continuous gradient decrease in Fe content from the top to the bottom along the thickness direction, with no abrupt concentration changes (Figure 2g). This validates the successful construction of a triple‐gradient structure in the FCFe‐G membrane, encompassing electrical conductivity, magnetic permeability, and pore size gradients.

### 2.2 Mechanical Robustness, Flexibility, and Environmental Stability of the FCFe‐G Multigradient Membrane

**Figure 3:**
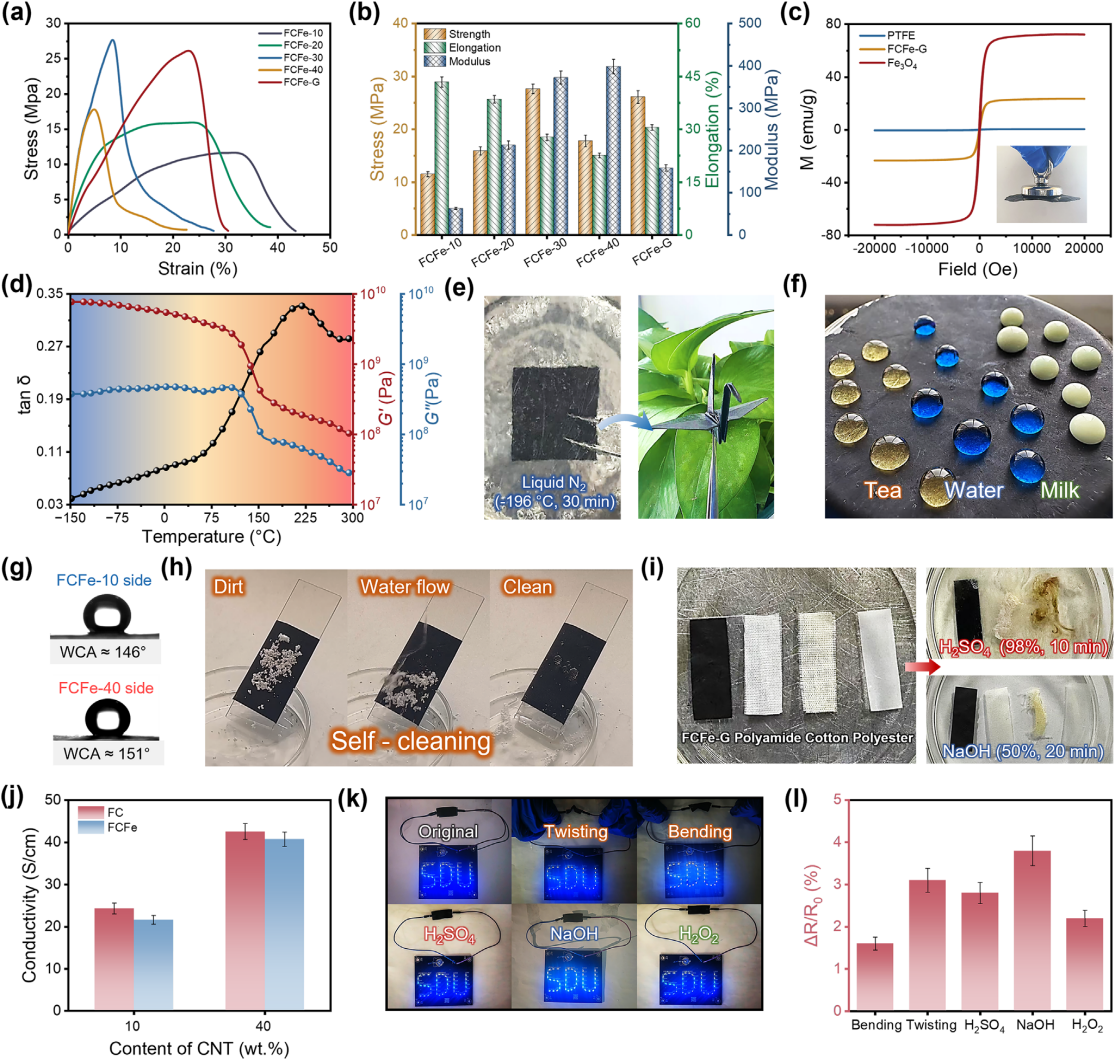
Characterization and macroscopic properties of the FCFe‐G membrane. a) Stress–strain curves of FCFe and FCFe‐G membranes. b) Comparison of tensile strength, elongation at break, and Young's modulus between FCFe and FCFe‐G membranes. c) Magnetic hysteresis loops of FCFe and FCFe‐G membranes (The illustration shows a magnet attracting the FCFe‐ membrane). d) Photograph of a folded FCFe‐G membrane shaped into an origami crane after liquid nitrogen treatment. e) DMA curves of the FCFe‐G membrane. f) Non‐wetting behavior of various liquid droplets on the FCFe‐G surface. g) WCAs measured on both sides of the FCFe‐G membrane. h) Self‐cleaning performance of the FCFe‐G membrane. i) Corrosion resistance comparison between FCFe‐G membranes and commercial textiles. j) Electrical conductivity on both sides of the gradient FCFe‐G membrane. k) The digital images of the luminance variation for the small bulbs tested. l) Resistance stability of the FCFe‐G membrane under simulated extreme environments.

In flexible wearable technologies, materials must combine mechanical robustness with superior deformation adaptability to meet the complex mechanical demands posed by dynamic human activities. The FCFe‐G membrane achieves this dual requirement through its structurally stabilized dual‐nanofibrous network and intercomponent synergistic effects. As shown in **Figures** **3**a and S3a, Supporting Information, FCFe single‐layer membranes demonstrate markedly enhanced tensile strength relative to the pure PTFE film, a direct consequence of the mechanically interlocking between CNT fibers and PTFE fibrils. Notably, FCFe‐30 attains a peak tensile strength of 27.64 MPa through an optimal CNT/Fe_3_O_4_ loading ratio that preserves the fusion interfaces between PTFE fibrils.^[^ ^37^ ^]^ While CNT incorporation elevates the Young's modulus of FCFe‐40 by 5.3 times, the spatial confinement effect concurrently suppresses PTFE molecular chain mobility, leading to a reduction in elongation at break to 52% of that of FCFe‐10.^[^ ^31^, ^38^ ^]^ The multigradient structure of the FCFe‐G membrane effectively alleviates the intrinsic trade‐off between strength and toughness. On one hand, nanofiber pinning at multilayer interfaces establishes a continuous stress‐distribution network, effectively mitigating localized stress concentrations inherent to single‐layer high‐filler systems (Figure 3b).^[^ ^39^ ^]^ Simultaneously, cracks tend to propagate along the interfacial regions within the gradient membrane, forming a zigzag path that facilitates efficient dissipation of fracture energy during crack growth.^[^ ^40^ ^]^ As a result, the optimized FCFe‐G membrane exhibits a tensile strength of 26.1 MPa, an elongation at break of 30.52%, and a Young's modulus of 158.49 MPa (Table S2, Supporting Information).

The magnetic hysteresis loop measurements revealed the magnetically responsive properties of the FCFe‐G membrane. As demonstrated in Figure 3c and Figure S3b, Supporting Information, pure PTFE shows negligible magnetic activity, whereas both FCFe and FCFe‐G membranes display typical ferrimagnetic behavior similar to Fe_3_O_4_. Notably, as the CNT content increases from 10 to 40 wt.%, the saturation magnetization of the FCFe membrane gradually decreases from 31.32 to 11.48 emu·g^−1^, confirming the formation of a magnetic gradient that is inversely oriented relative to the electrical gradient within the FCFe‐G structure. The FCFe‐G membrane exhibits a saturation magnetization of 22.24 emu·g^−1^, and its excellent magnetic responsiveness enabled rapid and reversible manipulation under an external magnetic field. Furthermore, due to the regular molecular alignment of the PTFE matrix, strong C–F covalent bonds, and its high molecular weight, the FCFe‑G membrane exhibits robust short‐term thermomechanical stability from −150 to 250 °C (well below the decomposition temperature of PTFE), with its storage modulus varying by less than two orders of magnitude (Figure 3d).^[^ ^17^ ^]^ As shown in Figure 3e, the FCFe‑G membrane remains foldable into intricate 3D configurations even after 30 min of liquid nitrogen treatment (−196 °C), validating its outstanding mechanical durability under extreme temperature conditions.

Outstanding hydrophobicity and corrosion resistance are critical for ensuring the long‐term stability of flexible wearable electronics in harsh environments. Benefiting from PTFE's low surface energy and the copper mesh‐assisted annealing‐induced surface microstructures, the FCFe‐G membrane exhibits exceptional water resistance.^[^ ^31^, ^41^ ^]^ As shown in Figure 3f,g, liquids such as water, tea, and milk exhibit pronounced non‐wetting behavior on the FCFe‐G surface, with the FCFe‐40 side achieving a water contact angle of 151°, which is indicative of superhydrophobicity. This superhydrophobicity endows the FCFe‐G with a unique self‐cleaning capability, where water droplets roll off effortlessly, effectively removing contaminants without leaving any residue (Figure 3h and Video S1, Supporting Information). Furthermore, the intrinsic chemical inertness of PTFE and CNT imparts the FCFe‐G membrane with remarkable corrosion resistance.^[^ ^36^ ^]^ As illustrated in Figure 3i, under strong acid and alkaline conditions, conventional textiles (cotton, polyester, and nylon) exhibit varying degrees of degradation, whereas the FCFe‐G membrane had remained structurally intact with no visible signs of corrosion.

Theoretically, the EMI shielding performance of CPCs is governed by both electrical conductivity and internal microstructure. Benefiting from the randomly oriented distribution of CNT within the PTFE nanofibrous network, the FCFe membranes progressively develop interconnected conductive pathways. As illustrated in Figure S4, Supporting Information, the electrical conductivity of the FCFe membrane increases from 3.45 to 52.26 S·cm^−1^ as the CNT content increases from 10 to 40 wt.%. In contrast, the FCFe‐G membrane, fabricated via the LBL assembly strategy, displays a smaller conductivity difference of only 27.56 S·cm^−1^ between its two sides (Figure 3j). This non‐abrupt conductivity gradient facilitates optimized impedance matching between individual layers. Compared to FC‐G, FCFe‑G exhibits slightly reduced electrical conductivity, which may be attributed to the electron transport hindrance caused by the non‐conductive magnetic fillers.^[^ ^42^ ^]^ Furthermore, the exceptional flexibility and durability of the composite membrane synergistically endow FCFe‐G with outstanding conductive stability. As demonstrated in Figure 3k,l, the LED's brightness remains virtually unchanged even after repeated bending, folding, or exposure to solvents, with resistance fluctuations kept below 4%. The superior hydrophobicity, stability, and robustness of FCFe‐G wearable membranes enable their reliable application in extreme environments involving mechanical deformation, high humidity, or chemical corrosion.

### 2.3 EMI Shielding Performance of the FCFe‐G Multigradient Membrane

**Figure 4:**
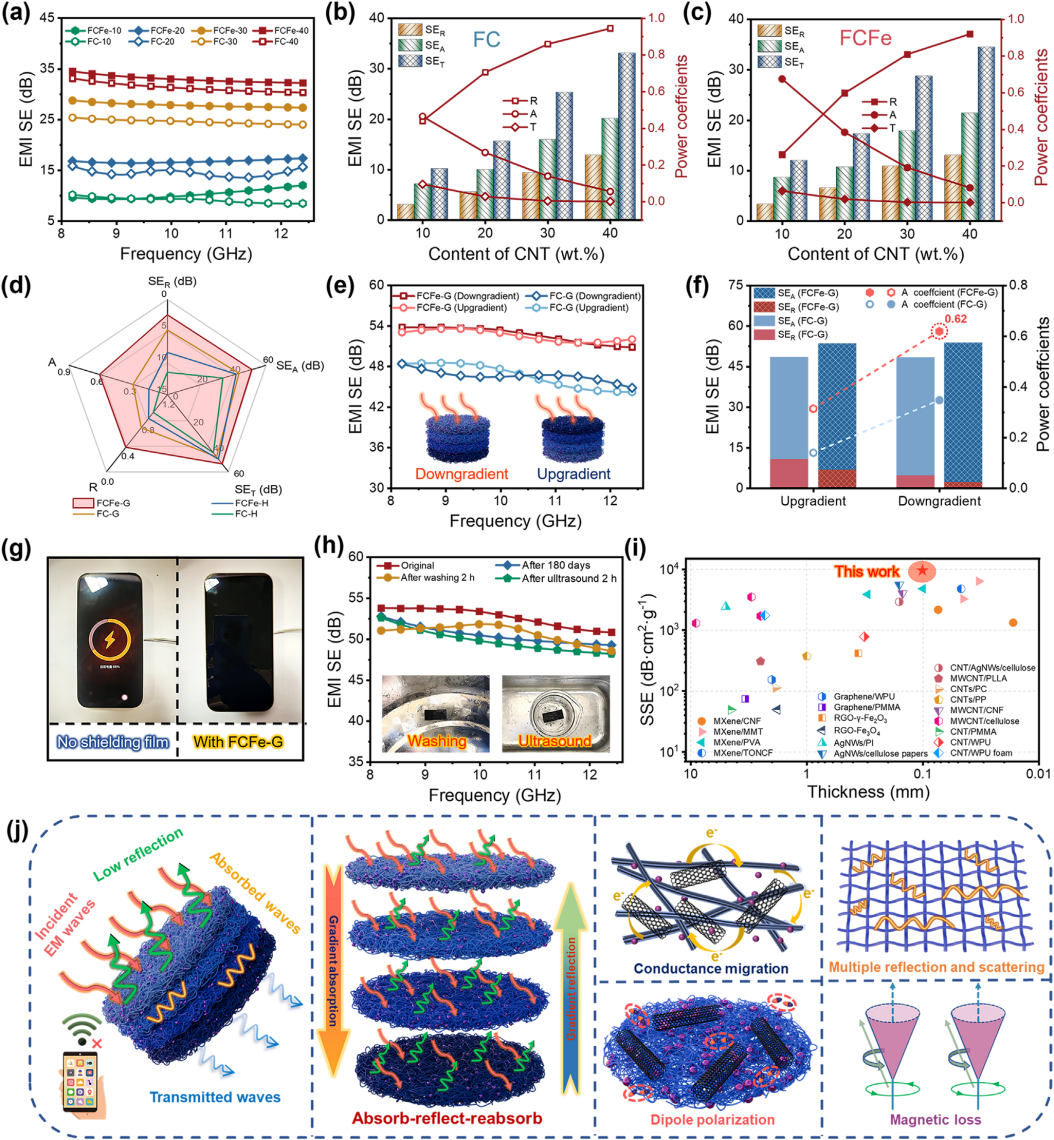
EMI shielding performance of FCFe and FCFe‐G membranes. a) EMI SE of FC and FCFe membranes with varying CNT contents. EMI shielding efficiency (SE_T_, SE_A_, and SE_R_) and power coefficients (R, T, and A) of b) FCFe and c) FC membranes. d) Comparative EMI shielding efficiency and power coefficient of FCFe‐G, FCFe‐H, FC‐G, and FC‐H membranes. e) EMI shielding efficiency of FC‐G and FCFe‐G membranes under EMW incidence from different directions. f) Directional dependence of the EMI shielding performance of FC‐G and FCFe‐G membranes. g) Photograph illustrating the FCFe‐G membrane effectively blocking wireless signal transmission. h) Environmental stability of the EMI shielding performance of the FCFe‐G membrane. i) Comparison of SSE and thickness of the FCFe‐G membrane with typical shields reported. j) Schematic illustration of the EMI shielding mechanism of the FCFe‐G membrane.

Benefiting from the continuous distribution of CNT conductive networks, the ordered alignment of Fe_3_O_4_ nanoparticles, and the internal porous structure, the FCFe single‐layer membrane achieves effective EMI shielding through an EM coupling loss mechanism. As shown in **Figure** **4**a, increasing the CNT content from 10 to 40 wt.% enhances the EMI SE of the FCFe membrane from 9.6 dB to 34.5 dB. Notably, due to the magnetic loss introduced by Fe_3_O_4_ nanoparticles, the FCFe membrane exhibits a 2–15% improvement in EMI SE compared to that of the FC membrane at the same CNT loading. Further analysis of the EM attenuation mechanism reveals that both absorption loss (SE_A_) and reflection loss (SE_R_) increase with increasing CNT content, with SE_A_ consistently accounting for over 60% of the total shielding efficiency (SE_T_) (Figure 4b,c). However, a high absorption ratio does not necessarily mean that the FCFe single‐layer membrane adopts an absorption‐dominated mechanism.^[^ ^42^ ^]^ Quantitative analysis of the shielding coefficients shows that once CNT content exceeds 10 wt.%, the absorption coefficient (A) decreases significantly from 0.675 to 0.384 and eventually reaches a minimum of 0.08 for the FCFe‐40 membrane. Moreover, the FC membranes exhibit even lower SE_A_ and A values. These findings indicate that although the incorporation of magnetic fillers and the design of porous structures enhance EMW absorption, the densification of the conductive network leads to severe surface impedance mismatch. Consequently, reflection remains the dominant shielding mechanism in the single‐layer membrane.

The aforementioned studies suggest that developing EMI shielding materials with both high shielding efficiency and superior anti‐reflection properties requires further optimization of the material's EM parameters and structural design to construct a cross‐dimensional gradient structure featuring continuous impedance transitions. To validate the efficacy of the multimodal gradient design, this work comparatively investigated four types of membranes: homogeneous conductive membrane (FC‐H), single electrical conductivity gradient membrane (FC‐G), homogeneous EM membrane (FCFe‐H), and EM multigradient membrane (FCFe‐G). The experimental findings demonstrate that, among all samples, the FCFe‐G membrane exhibits optimal shielding performance, achieving a maximum EMI SE of 53.79 dB—well above the commercial threshold (>20 dB) (Figure S5a, Supporting Information). Further investigation into the anti‐reflection capability reveals that the SE_R_ of the FCFe‐G membrane is as low as 2.43 dB, which is 9.05, 5.92, and 2.38 dB lower than that of the FC‐H, FC‐G, and FCFe‐H membranes, respectively (Figure 4d). Notably, FCFe‐G is the only membrane with an A coefficient (0.62) exceeding its reflection (R) coefficient (0.38), indicating a truly absorption‐dominated shielding mechanism. Such outstanding performance stems from the precisely engineered cross‐dimensional gradients in the FCFe‐G membrane. Specifically, an ascending electrical conductivity gradient (bottom to top), coupled with a descending magnetic permeability gradient (top to bottom), effectively minimizes impedance mismatches at successive interfaces, thereby suppressing EMW reflection. Simultaneously, the multilayer pore size gradient design not only increases the propagation path length of EMWs within the material, thereby enhancing energy dissipation, but also enables gradual impedance matching through the transition from larger to smaller pores. This hierarchical structure reduces the reflection between the incident waves and the material surface. As a result, FCFe‐G delivers ultrahigh SE_T_ while maintaining exceptional anti‐reflection performance.

The subsequent investigation focused on the EMI shielding performance under different EMW incidence directions. As depicted in Figure 4e,f, the direction from the low‐conductivity layer to the high‐conductivity layer is labeled “Downgradient,” whereas the reverse direction is labeled “Upgradient.” For both FC‐G and FCFe‐G gradient membranes, SE_T_(Downgradient) and SE_T_(Upgradient) values are nearly identical; however, SE_A_(Downgradient) exceeds SE_A_(Upgradient) by 4.73 dB. This indicates that while the incident direction has a negligible impact on the overall shielding performance, it significantly influences the EMW attenuation mechanisms. ​Further analysis of the loss factors reveals that the A(Upgradient) coefficient of the FCFe‐G is 0.31, which is only 50% of that under “Downgradient” incidence. These discrepancies in loss factors primarily arise from the asymmetric impedance matching and the directional dynamics of multilayer EM energy dissipation intrinsic to the FCFe‐G gradient structure, as elaborated below: 1. When EMWs are incident from the FCFe‐10 layer (low conductivity, high permeability, large pores), optimal impedance matching is achieved at the air–film interface, thereby facilitating efficient entry of EMWs with minimal reflection. Thus, the uppermost FCFe‐10 layer functions as an “absorption layer,” resulting in a low SE_R_ value. 2. The bottom high‐conductivity FCFe‐40 layer acts as an effective “reflection layer,” effectively blocking the transmission of residual EMWs and contributing to a high SE_T_. 3. The middle layers within the FCFe‐G membrane constitute a “dissipation zone” through the coupled conductivity‐permeability‐pore size gradient. The unique cross‐dimensional gradient design establishes continuous impedance gradients within the material, thereby enabling sequential attenuation of EM energy through the synergistic interplay of dielectric and magnetic losses.^[^ ^43^ ^]^ Therefore, the FCFe‐G membrane efficiently attenuates EMWs via an “absorption–reflection–reabsorption” mechanism, while substantially mitigating secondary radiation effects.

Notably, EMI SE results indicate that the FCFe‐G multigradient membrane is capable of blocking up to 99.996% of incident EMWs. Consequently, even at a minimal thickness of 101.1 µm, the FCFe‐G membrane effectively disrupts wireless signal transmission and terminates charging processes (Figure 4g). In practical applications, EMI shielding materials must retain stable performance under harsh environmental conditions. As shown in Figure 4h, the FCFe‐G membrane exhibited only a 1.65 dB reduction in SE_T_ after 180 days of storage at room temperature, which can be attributed to the intrinsic anti‐aging characteristics of CNT and the PTFE matrix. More importantly, the membrane demonstrates excellent resistance to mechanical fatigue, maintaining 97.8% and 95.1% of its initial SE_T_ after 2 h of ultrasonic treatment and vigorous rinsing, respectively. Furthermore, the FCFe‐G membrane retains robust shielding performance under acidic, alkaline, and extreme temperature conditions, with SE_T_ attenuation remaining below 8% in all cases (Figure S5b, Supporting Information). To quantify the advantages of the film‐based shielding device, the SSE value was further calculated to evaluate the synergistic balance between lightweight design and high‐performance EMI attenuation (Figure 4i). The results reveal that the FCFe‐G membrane achieves an exceptional SSE value of 9539.52 dB·cm^2^·g^−1^, surpassing many previously reported representative EMI shielding materials, including those based on CNT, graphene, MXene, and metal films (Table S3, Supporting Information).

To systematically elucidate the EMI shielding mechanism of the FCFe‐G membrane, the attenuation pathways of EMWs are schematically illustrated in Figure 4j. When EMWs are incident along the upgradient, the shielding behavior is primarily governed by strong reflection induced by the highly conductive FCFe‐40 layer, resulting in a high R and substantial SE_R_. In contrast, when EMWs are incident along the downgradient, the FCFe‐10 absorption layer—with its low electrical conductivity, high magnetic permeability, and large pore size—facilitates effective impedance matching with free space, thereby enabling greater EMW penetration into the gradient structure. Within the interior of the FCFe‐G membrane, the hierarchical multigradient structure continuously optimizes impedance matching across layers, minimizing interfacial reflections and allowing EMWs to traverse the multilayer nanofibrous network sequentially. During propagation, EMWs interact with the high‐density free charge carriers in the conductive CNT network, leading to significant conduction loss and the conversion of EM energy into thermal energy.^[^ ^44^ ^]^ Meanwhile, the hierarchical porous structure formed by the dual‐nanofibrous network promotes multiple internal reflections and scattering events, extending the effective propagation path of EMWs and enhancing absorption.^[^ ^45^ ^]^ Additionally, the heterogeneous interfaces between PTFE, CNT, and Fe_3_O_4_ nanoparticles generate localized dipoles due to asymmetric charge distributions. These dipoles dynamically polarize under the alternating EM field, contributing to enhanced dielectric loss via interfacial and dipolar polarization.^[^ ^46^ ^]^ Furthermore, the uniformly dispersed magnetic Fe_3_O_4_ nanoparticles introduce considerable magnetic loss through natural resonance and eddy current dissipation mechanisms.^[^ ^22^ ^]^ As the residual EMWs reach the bottom FCFe‐40 layer, a severe impedance mismatch induces strong reflection, redirecting the waves back toward the upper absorption region and forming an efficient “absorption–reflection–reabsorption” cyclic attenuation loop.^[^ ^43^ ^]^ This multistage dissipation pathway enables the FCFe‐G membrane to realize a synergistic combination of robust EMI shielding and low reflection through a cascade mechanism involving “impedance matching–multilevel polarization—reabsorption.” These findings provide a novel theoretical foundation and structural paradigm for the rational design of next‐generation multigradient‐structured EMI shielding materials that are lightweight, tunable, and highly absorptive.

### 2.4 Visualized EMI Shielding Simulation of the FCFe‐G Multigradient Membrane

**Figure 5:**
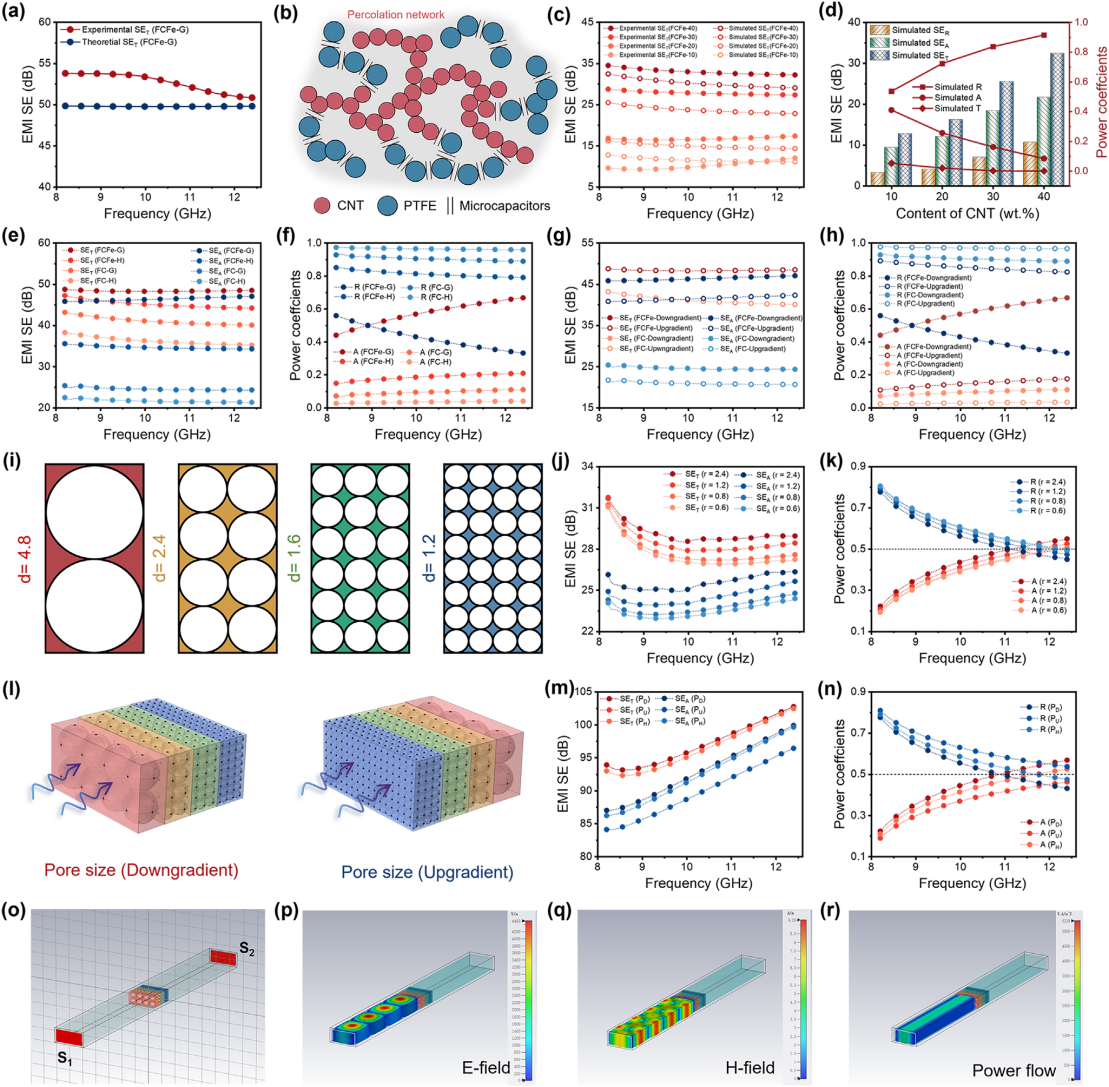
Theoretical and simulated EMI shielding performance of FCFe and FCFe‐G membranes. a) Experimentally tested SE_T_ and theoretically calculated SE_T_ of a homogeneous shield. b) Schematic illustration of microcapacitor formation within the FCFe‐G membrane. c) Simulated SE_T_ of FCFe membranes with varying CNT contents. d) Simulated EMI SE and power coefficients of FCFe membranes with different CNT contents. e) Simulated EMI SE and f) power coefficients of FC‐G, FC‐H, FCFe‐G, and FCFe‐H membranes. g) Simulated EMI SE and h) power coefficients of FCFe‐G and FC‐G under EMW incidence from different directions. i) Side‐view models of membranes with identical porosity but varying pore size. j) Simulated EMI SE and k) power coefficients of single‐layer membranes with different pore size. l) Schematic of the pore‐gradient model under EMW incidence from different directions. Effect of incident direction on the m) EMI SE and n) power coefficient of the pore size gradient model. o) Schematic of pore‐gradient model in CST simulation. p) Electric field, q) magnetic field, and r) power flow density distributions of the model under EMW incidence along the P_D_ direction.

To further elucidate the EMI shielding mechanism of the multigradient structure, a combined strategy of theoretical calculations and finite element simulations was employed to decouple the respective contributions of intrinsic EM parameters and multilayer porous structures to EMW absorption. As shown in **Figures** **5**a and S6, Supporting Information, when considering only the intrinsic electrical conductivity and thickness of the material, the theoretical SE_T_ of the FCFe‐G membrane was calculated to be merely 49.84 dB, which is substantially lower than the experimentally measured SE_T_ (S1, Supporting Information).^[^ ^47^, ^48^ ^]^ This substantial discrepancy underscores the critical role of insulating structures beyond the conductive network in enhancing overall EMI shielding performance. As shown in Figure 5b, while some CNTs form a percolative conductive network, a considerable portion of the conductive fillers remains isolated or poorly connected within the PTFE matrix. These isolated conductive elements, together with the surrounding polymer, constitute numerous pseudo‐parallel plate microcapacitors, where the conductive fillers act as polarizable electrodes and the PTFE matrix serves as the dielectric layer. Upon exposure to incident EM waves, these microcapacitor structures strongly couple with the EM field, resulting in enhanced interfacial polarization and dipolar relaxation.^[^ ^49^ ^]^

Subsequently, 3D EM models were constructed using CST Microwave Studio to quantitatively analyze the role of electrical–magnetic gradient structures in optimizing shielding performance (S2, Supporting Information). Simulations of FC and FCFe single‐layer membranes (excluding microstructural features) revealed a positive correlation between SE_T_ and CNT content, which was consistent with the experimental results (Figure 5c and Figure S7a, Supporting Information). However, the simulated SE_T_, SE_A_, and A were all substantially lower than their experimental counterparts, likely due to the omission of internal reflection and scattering effects within the dual‐nanofibrous structure (Figure 5d and Figure S7b, Supporting Information). Further comparative investigations between homogeneous and gradient structures revealed that the FCFe‐G membrane, featuring optimized electrical–magnetic dual gradients, exhibited superior EMI shielding performance, with simulated SE_T_ of 48.49 dB, SE_A_ of 45.84 dB, and an A value of 0.66 (Figure 5e,f). Notably, reversing the positions of the excitation (S_1_) and detection (S_2_) sources led to a negligible change in EMI SE, but caused a significant reduction in SE_A_ by 4.74 dB and decreased the A value to only 25.76% of its original level (Figure 5g,h). This directional asymmetry conclusively validates the pivotal role of gradient orientation design in realizing absorption‐dominant shielding behavior, consistent with experimental data. Finally, EM field distribution simulations were performed to visualize shielding differences across distinct structural configurations (Figure S8, Supporting Information). As shown in Figure S9, Supporting Information, the pure PTFE film exhibited negligible attenuation of both electric and magnetic fields due to the absence of conductive and magnetic components. The FCFe‐10 membrane permitted partial EM wave penetration from S_1_ to S_2_, whereas FCFe‐40 with 40% CNTs induced substantial field suppression at S_2_ via strong conductive loss. The FCFe‐G gradient membrane surpassed single‐layer systems, achieving near‐total EM energy dissipation through synergistic electric–magnetic gradient effects. Furthermore, FCFe and FCFe‐G membranes exhibit responses to both electric and magnetic fields, confirming that EMW attenuation mechanisms involve not only conductive losses (reflection and absorption) but also magnetic losses.^[^ ^8^, ^21^ ^]^

In addition to EM gradient engineering, hierarchical pore structure emerges as equally critical yet underexplored determinants of EMI shielding performance. To exclusively probe the effect of the pore size gradient in the FCFe‐G membrane, it was parametrically isolated via COMSOL modeling while maintaining identical EM parameters. By varying pore radius, the EMI shielding performance of single‐layer porous structures with uniform pore size was simulated (Figure 5i). As shown in Figure 5j, reducing the pore size from 4.8 to 1.2 mm resulted in only a 3.47% decrease in SE_T_, while SE_A_ exhibited a more pronounced reduction of 8.69%. Furthermore, as the average pore size decreased, the A coefficient declined from 0.55 to 0.49, representing a reduction of over 10% (Figure 5k). These findings highlight the pivotal role of internal pore dimensions in governing EMW absorption. On one hand, larger pores modulate the effective dielectric constant to better match free‐space impedance.^[^ ^50^, ^51^ ^]^ On the other hand, they promote multiple internal reflection and scattering events, thereby prolonging the propagation path of EMWs within the material and amplifying energy dissipation.^[^ ^52^, ^53^ ^]^

Based on the aforementioned simulation results, a four‐layer porous structure was further configured with a graded arrangement of pore size to replicate the actual structure of the FCFe‐G membrane. As shown in Figure 5l, the descending pore size gradient (from large‐pore to small‐pore layers), ascending pore size gradient (inverse direction) and homogeneous porous structure were defined as “P_D_,” “P_U_,” and “P_H_,” respectively. While the SE_T_ remained nearly identical for EMW incidence along the P_D_, P_U_, and P_H_, the SE_A_(P_D_) reached 99.89 dB, which is 3.47 dB higher than SE_A_(P_U_), due to asymmetric impedance matching at both ends of the pore size gradient configuration (Figure 5m). This directional mismatch further amplifies the absorption advantage, with A(P_D_) exceeding those of A(P_H_) and A(P_U_) by 7.65% and 21.79%, respectively (Figure 5n). Moreover, visualization simulations demonstrate that when EMWs are incident along P_D_, the hierarchical pore size gradient structure nearly eliminated the EM field and power density at the receiving end, validating its near‐complete EM energy trapping capability (Figure 5o–r). Therefore, this multigradient design paradigm establishes a deterministic bridge between micro–nano structure control and macroscopic EM response, thereby enabling an advanced design paradigm for absorption‐priority shielding materials.

### 2.5 Anisotropic Thermal Conductivity and Fire Warning Performance of FCFe‐G Multigradient Membranes

**Figure 6:**
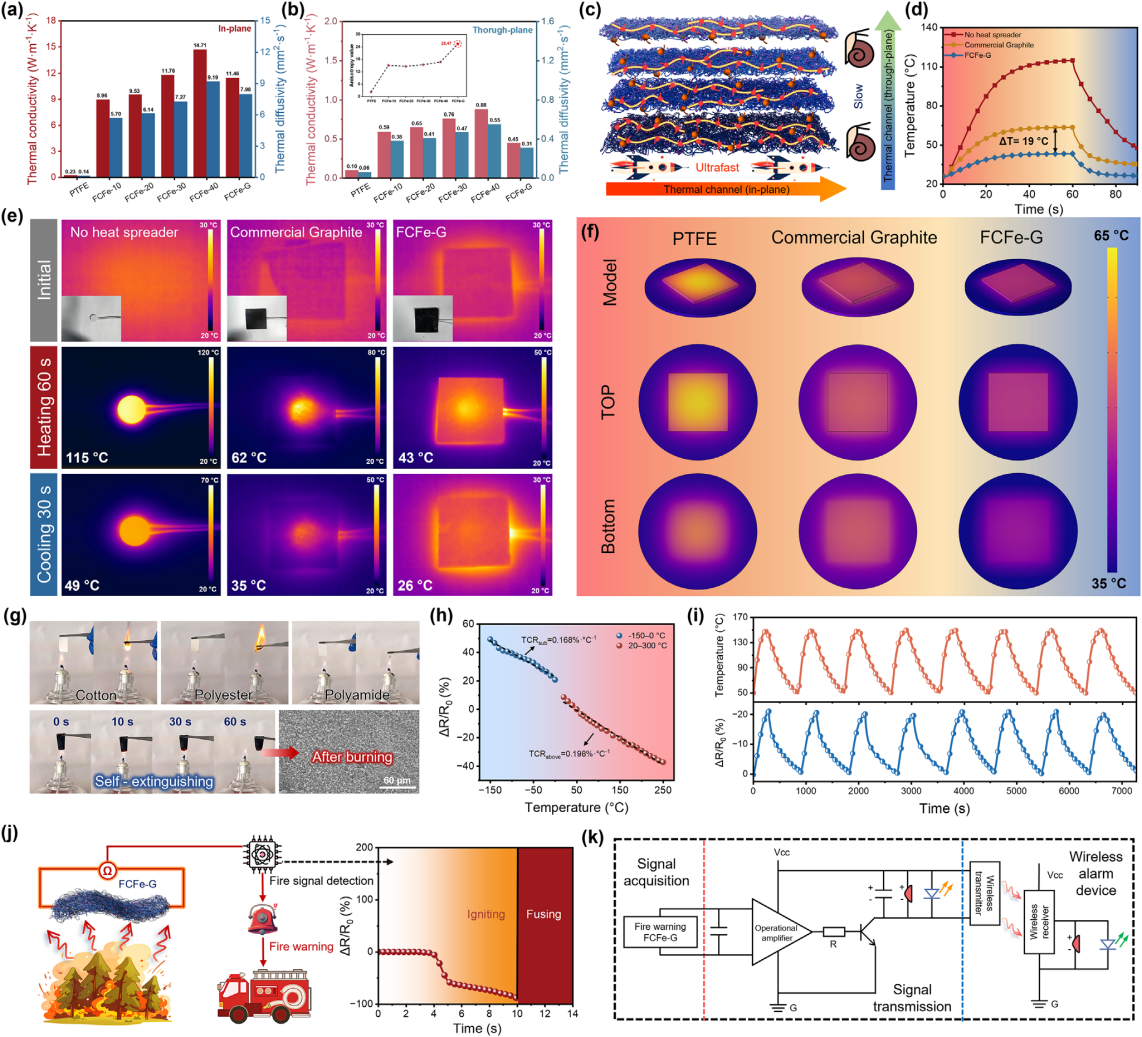
Anisotropic thermal conductivity and fire‐sensing capabilities. a) In‐plane thermal conductivity and thermal diffusivity of FCFe and FCFe‐G membranes. b) Through‐plane thermal conductivity and thermal diffusivity of FCFe and FCFe‐G membranes (inset: anisotropy ratio). c) Schematic illustration of the anisotropic heat conduction mechanism in the FCFe‐G membrane. d) Heating and cooling curves of the heat source under the three conditions. e) Infrared thermographic images of the heat source under the three conditions. f) Simulation results comparing the thermal dissipation of PTFE, commercial graphene, and FCFe‐G membranes. g) Comparison of flame‐retardant performance between the FCFe‐G membrane and commercial textiles. h) Relative resistance variation of the FCFe‐G thermistor. i) Cyclic heating/cooling cycles of the FCFe‐G membrane. j) Fire alarm mechanism based on the FCFe‐G membrane. k) Circuit logic diagram of the wireless fire‐alarm device.

The highly integrated design of electronic devices not only induces severe EMI but also leads to inevitable heat accumulation within confined spaces, significantly compromising the service reliability and lifespan of components.^[^ ^54^ ^]^ Therefore, EMI shielding materials for flexible and wearable electronics must possess superior thermal dissipation capabilities. Systematic evaluations of thermal conductivity and diffusivity were conducted on PTFE, FCFe, and FCFe‐G membranes.^[^ ^55^ ^]^ As shown in **Figure** **6**a, the in‐plane thermal conductivity and diffusivity of FCFe single‐layer membranes increased with CNT content, reaching maximum values of 14.71 W·m^−1^·K^−1^ and 9.19 mm^2^·s^−1^ at FCFe‐40. This enhancement is attributed to the formation of an interconnected dual‐nanofibrous network between the CNT and PTFE, which facilitates efficient phonon transport.^[^ ^32^ ^]^ Impressively, the FCFe‐G multigradient membrane also exhibited excellent in‐plane thermal performance, with in‐plane thermal conductivity and diffusivity values of 11.46 W·m^−1^·K^−1^ and 7.98 mm^2^·s^−1^, which are 49.8 and 57.1 times higher than those of pure PTFE membrane. In sharp contrast to the significantly enhanced in‐plane thermal transport performance, the through‐plane thermal conductivity and diffusivity of the FCFe‐G membrane remain low, at 0.45 W·m^−1^·K^−1^ and 0.31 mm^2^·s^−1^, respectively, resulting in a high anisotropy ratio of 25.47 (Figure 6b). This pronounced anisotropy stems from the in‐plane alignment of conductive CNT nanofibers and the cascaded interfacial thermal resistance between multilayered structures. As illustrated in Figure 6c, the LBL assembly process drives the horizontally aligned arrangement of interconnected CNT/PTFE/Fe_3_O_4_ nanofibrous network, establishing ultrafast in‐plane phonon transport pathways while minimizing scattering.^[^ ^56^ ^]^ This structure enables rapid lateral heat flux dissipation from hotspots to edges, substantially enhancing in‐plane thermal conductivity. Conversely, the cascaded interfacial thermal resistance and discontinuous through‐plane nanofibrous structure induce multiscale phonon scattering at interlayer interfaces, leading to significant energy dissipation during vertical heat transport.^[^ ^57^, ^58^ ^]^

To assess the practical thermal management capability of the FCFe‐G composite membrane, a high‐temperature ceramic heating plate was employed as the heat source. The heat dissipation material was integrated onto the top surface of the heat source, and its thermal response was comparatively evaluated under three configurations: without a heat spreader, with the commercial graphene heat spreader, and with the FCFe‐G membrane. As illustrated in Figure 6d,e, during 60 s of continuous heating, the surface temperature of the heat source without any dissipation material surged sharply from 25.9 to 115.1 °C. The commercial graphene heat spreader significantly reduced the peak temperature to 63.5 °C, while the FCFe‐G membrane demonstrated even greater thermal regulation capability, limiting the maximum temperature to 43.3 °C. Moreover, after 30 s of natural cooling following power‐off, the surface temperature of the FCFe‐G membrane dropped to 26.2 °C, which is markedly lower than that of the bare heat source (48.6 °C) and the commercial graphene spreader (35.2 °C). Infrared thermography further confirmed that FCFe‐G provided a more uniform in‐plane temperature distribution in comparison with the commercial counterpart, thus directly validating its exceptional heat‐spreading efficiency.

To further investigate the heat transport dynamics, COMSOL Multiphysics simulations were conducted to model the thermal conduction processes of pure PTFE, commercial graphene, and the FCFe‐G composite membranes (Figure 6f). By positioning the heat source on the top surface of each sample, the transient thermal response—particularly the temperature distribution across the top and bottom surfaces—was systematically analyzed (S3, Supporting Information). Simulation results demonstrated that under identical heat flux inputs, the central region of FCFe‐G consistently maintained lower temperatures than the other two films, corroborating its ultrahigh in‐plane thermal conductivity. This finding aligns precisely with practical thermal management requirements. In high‐power electronics, heat spreaders typically exhibit lateral dimensions significantly exceeding hotspot areas, rendering in‐plane thermal conductivity the critical determinant of cooling efficiency.^[^ ^59^ ^]^ Overall, the FCFe‐G membrane achieves dual functionality through directional heat transport mechanisms. On the one hand, it rapidly dissipates heat from hotspots along the in‐plane direction to prevent localized overheating and device failure.^[^ ^60^ ^]^ On the other hand, its intrinsically low through‐plane thermal conductivity serves as a thermal barrier, minimizing heat transfer to underlying electronics or human skin.^[^ ^61^ ^]^

Many conventional CPCs are plagued by high flammability, posing severe fire risks in EMI shielding and thermal management applications.^[^ ^62^ ^]^ Consequently, the fire safety of the FCFe‐G membrane is critical for its practical deployment. Vertical burning tests reveal that the FCFe‐G membrane withstands sustained ignition for 60 s of continuous alcohol‐lamp flame exposure, emits only minimal smoke, and self‐extinguishes within 5 s after the flame is removed (Figure 6g and Video S2, Supporting Information). In stark contrast, commercial textiles ignite vigorously within 5 s, accompanied by rapid flame propagation. This exceptional self‐extinguishing behavior is attributed to a dual mechanism of flame retardancy. First, the inherent fire resistance of the PTFE matrix and CNT nanofibers plays a pivotal role.^[^ ^31^ ^]^ Second, the formation of a dense amorphous carbon barrier during combustion effectively impedes flame penetration and heat transfer.^[^ ^32^ ^]^

Notably, the intrinsic negative temperature coefficient behavior of CNT, combined with the dual‐nanofibrous network's efficient thermal and electrical pathways, endows the FCFe‐G membrane with exceptional temperature‐sensing capabilities. As shown in Figure 6h, the temperature‐normalized resistance curve exhibits a linear response across two broad temperature intervals (−150 to 0 °C and 20 to 250 °C), with closely matched temperature coefficients of resistance (TCR): TCR_sub_ = 0.168% °C^−1^ and TCR_above_ = 0.196% °C^−1^. In addition, the thermal index of this FCFe‐G membrane‐based thermistor is calculated to be ≈197 K. As shown in Figure 6i, cyclic heating/cooling experiments in the 50–150 °C range further validate the thermistor performance of the FCFe‐G membrane, where the ΔR/R_0_ curve exhibits precise synchronization with temperature fluctuations across eight consecutive cycles without noticeable signal degradation, confirming its high sensitivity, accuracy, and excellent operational stability for temperature sensing applications. Leveraging the synergistic advantages of flame‐retardant properties and temperature‐sensing capabilities, a wireless fire‐alarm system was developed based on the FCFe‐G membrane (Figure 6j). During fire incidents, the sensor resistance exhibits a two‐stage response: an initial sharp decline in ΔR/R_0_ (temperature‐sensitive zone), followed by a rapid rise approaching infinity near the material's thermal decomposition threshold (failure‐alarm zone). The system's logic design is illustrated in Figure 6k, where a microcontroller continuously monitors the resistance signal and amplifies it using an operational amplifier. When the signal exceeds a predefined threshold, a wireless transmission device sends instructions to the receiver, triggering an audible or visual alarm and activating emergency evacuation protocols.^[^ ^62^ ^]^ This integrated design provides an innovative solution for early fire detection and rapid evacuation.

### 2.6 The Electro/Photothermal Conversion Performance of FCFe‐G Multigradient Membrane

**Figure 7:**
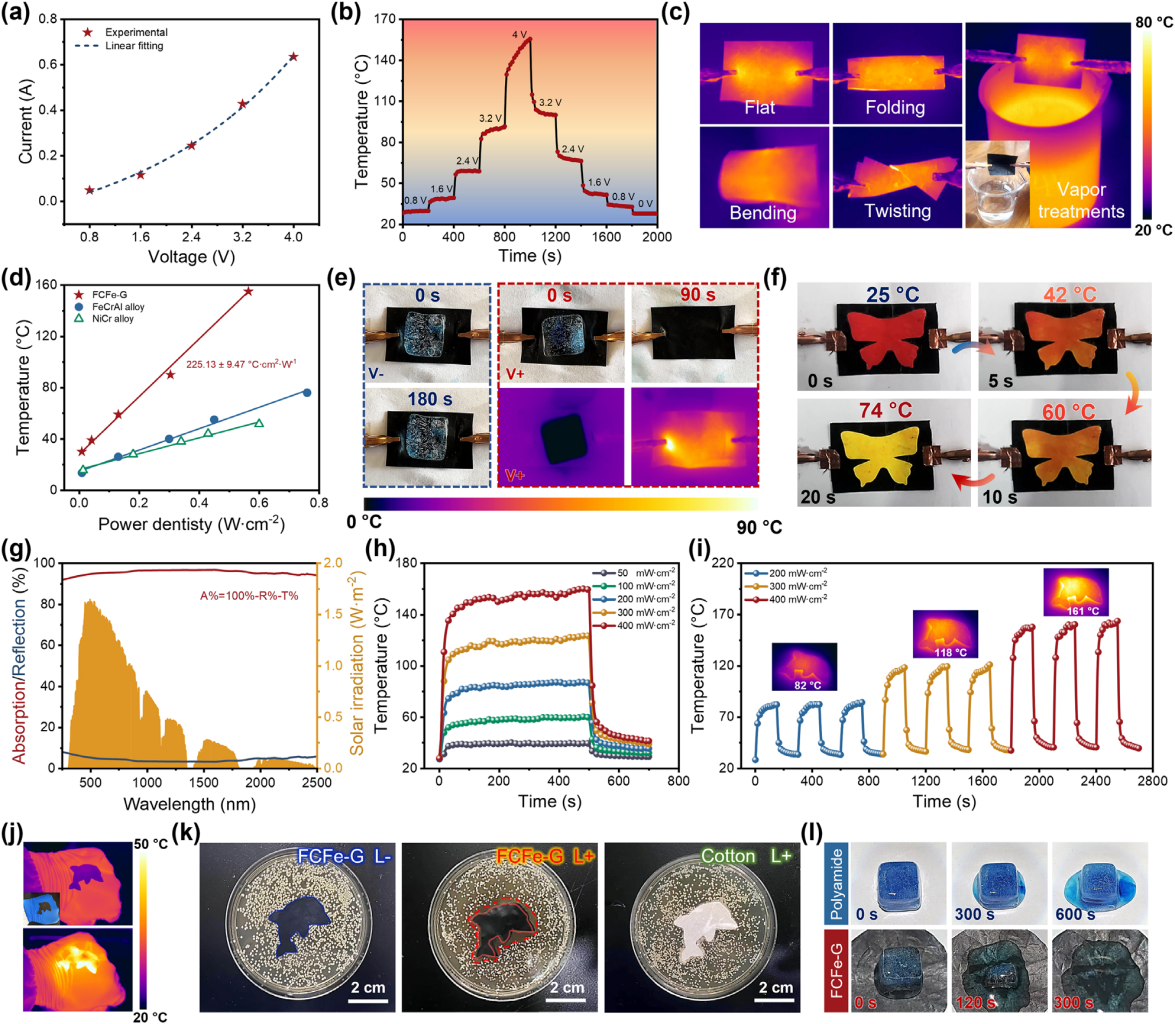
Electro/photothermal conversion properties of FCFe‐G membrane. a) I–V characteristics of the FCFe‐G membrane. b) Temperature variation curves of the FCFe‐G membrane at different voltages. c) Infrared images of FCFe‐G during mechanical deformations and steam exposure. d) Comparison of electrothermal efficiency between the FCFe‐G membrane and other commercial heaters. e) Optical and infrared images during the electrothermal de‐icing process. f) Thermochromic behavior of the FCFe‐G membrane. g) UV–vis–NIR absorption and reflection spectra of the FCFe‐G membrane. h) Temperature–time variation curves of the FCFe‐G membrane under simulated solar irradiation. i) Photothermal cycling stability under different light power densities. j) Demonstration of thermal therapy performance for wearable applications. k) Photothermal antibacterial performance of the FCFe‐G membrane against Staphylococcus aureus. l) Photothermal de‐icing performance of the FCFe‐G membrane.

To ensure high EMI shielding efficiency in extreme environments, exceptional integrated heating capabilities are indispensable. Owing to its outstanding electrical and thermal conductivity, the flexible FCFe‐G membrane is identified as a compelling candidate for portable electrothermal devices. As shown in **Figure** **7**a, the surface voltage and current of the FCFe‐G membrane display a strong positive correlation, confirming its reliability as a high‐performance electrothermal heater. Dynamic heating tests under 0.8–4 V reveal that the FCFe‐G membrane offers controllable electrothermal performance, with equilibrium temperatures ranging from 30.4 to 156.9 °C (Figure S10a, Supporting Information). The surface temperature of the FCFe‐G membrane rapidly increases within 10 s and remains stable under continuous voltage input. Upon voltage removal, a sharp decline from the saturation temperature to room temperature is observed, highlighting the ultrafast response of FCFe‐G in electrically driven thermal conversion. Notably, even under a low voltage perturbation of 0.8 V, the composite membrane enables fast and stable temperature regulation, meeting the stringent sensitivity requirements of smart thermal management systems (Figure 7b).

To further explore the reliability and durability of the FCFe‐G membrane as an electrothermal conversion device, long‐term heating stability and cyclic heating/cooling tests are conducted. As depicted in Figure S10b,c, Supporting Information, the FCFe‐G membrane maintains a stable equilibrium temperature for over 3000 s under a constant voltage of 3.2 V, with uniform surface temperature distribution throughout the process. Moreover, it delivers consistent and repeatable thermal output across multiple voltage cycles ranging from 1.6 to 4 V. Remarkably, owing to its flexible yet durable dual‐nanofibrous structure and strong environmental tolerance, the FCFe‐G membrane maintains uniform and stable heat output under complex mechanical deformations (bending, folding, twisting) and harsh environments such as high‐temperature steam (Figure 7c). This makes it compatible with dynamic human motion for localized thermotherapy and precision temperature control in smart textiles. Compared to conventional metal, alloy, and carbon‐based heaters, FCFe‐G demonstrates a higher electrothermal conversion efficiency (H_p_ = 225.13 °C·m^2^·W^−1^), enabling elevated operational temperatures at equivalent power densities (Figure 7d and Figure S10d, Supporting Information).^[^ ^63^ ^]^ In addition to heating functionality, its intrinsic hydrophobicity and low‐voltage thermal activation capability enable efficient active deicing. As shown in Figure 7e, under a low operating voltage of 2.4 V, ice rapidly melts, slides off, and completely evaporates within 90 s, offering a promising anti‐icing solution for polar‐region equipment. Uniquely, the FCFe‐G membrane integrates real‐time visual temperature feedback. When stimulated at 3.2 V, its surface color‐changing layer exhibits a noticeable color change within 5 s, and the butterfly pattern completely transitions from red to yellow after 20 s of heating (Figure 7f). This fast and intuitive color‐to‐temperature mapping offers a dual‐safety mechanism for overheating alerts and intelligent thermal regulation, greatly enhancing operational safety and functional controllability in practical applications.

Benefiting from the broad‐spectrum light absorption characteristics and photoinduced electron‐phonon coupling effect of CNT, CNT‐based composites are capable of directly converting solar energy into thermal energy.^[^ ^64^ ^]^ Furthermore, the unique dual‐nanofibrous network of FCFe‐G membranes amplifies the light absorption, internal multiple scattering, and reflection within the porous network, thereby achieving a solar absorptance exceeding 90% across the solar irradiance spectrum, which lays a foundation for efficient photothermal conversion (Figure 7g).^[^ ^65^ ^]^ Under simulated sunlight irradiation, the surface temperature of the FCFe‐G membrane rapidly increases, and the equilibrium temperature exhibits a linear relationship with incident light intensity (Figure S11a, Supporting Information). At light power densities of 50, 100, 200, 300, and 400 mW·cm^−2^, the steady‐state temperatures reach 39.4, 59.7, 87.5, 123.2, and 159.7 °C, respectively (Figure 7h). Notably, the surface temperature of the FCFe‐G membrane responds rapidly to variations in light power density gradients, demonstrating controllable and reversible photothermal behavior (Figure S11b, Supporting Information). The durability of the FCFe‐G membrane as a photothermal material was further evaluated through accelerated light‐aging tests. As demonstrated in Figure 7i and Figure S11c, Supporting Information, the FCFe‐G membrane exhibits excellent photothermal stability, maintaining consistent heating–cooling profiles over repeated xenon lamp on/off cycles and sustaining steady‐state temperatures for over 2000 s under constant light intensity. Such robust photothermal behavior enables promising applications in flexible and wearable devices. For instance, at a low light power density of 100 mW·cm^−2^, the membrane generates a uniform thermal field of ≈48 °C across its surface, fulfilling adaptive thermal regulation requirements for outdoor garments (Figure 7j).

Leveraging its controllable photothermal properties, the FCFe‐G membrane also demonstrates effective photothermal antibacterial activity. As shown in Figure 7k, when a culture medium inoculated with Staphylococcus aureus was covered with the FCFe‐G membrane and irradiated under low light power density for 20 min, an efficient bacteriostatic zone was observed around the membrane due to its photothermal‐induced bactericidal effects.^[^ ^66^ ^]^ In contrast, no bacteriostatic zone was observed in either the non‐irradiated FCFe‐G membrane or the pure PTFE film. Furthermore, the synergistic effects of the superhydrophobic surface and photothermal conversion endow the FCFe‐G membrane with exceptional photothermal deicing capability. As demonstrated in Figure 7l, under simulated sunlight irradiation, ice deposited on the FCFe‐G membrane rapidly melts into water within 300 s and subsequently slides off the surface due to its ultralow adhesion. This performance starkly outperforms that of the bare substrate, which exhibits only partial melting even after 600 s, corresponding to an improvement of over 100% in deicing efficiency. Such rapid and energy‐efficient ice removal greatly expands its applicability in consumer electronics, extreme climate conditions, and aerospace thermal management systems.

## 3 Conclusion

In summary, lightweight and flexible FCFe‐G multifunctional composite membranes with multigradient structures were successfully fabricated via shear‐induced in situ fibrillation and a LBL assembly strategy, enabling exceptional EMI shielding performance. The synergistic interaction between intralayer interconnected dual‐nanofibrous networks and interlayer nanofiber pinning effects imparts the FCFe‐G membrane with a remarkable tensile strength of 27.14 MPa, while integrating superhydrophobicity, thermal stability, and chemical corrosion resistance. By simultaneously optimizing the hierarchical porous framework and EM filler distribution, the electro–magnetic–porous triple‐gradient structure minimizes impedance mismatch. Consequently, the FCFe‐G membrane, with a thickness of only 101.1 µm, achieves an EMI SE of 53.79 dB at a low R coefficient of 0.38 via the “absorption–reflection–reabsorption” EMI shielding mechanism. Notably, its SSE reaches an impressive 9539.52 dB·cm^2^·g^−1^, surpassing many conventional reflection‐dominated shielding materials. Moreover, owing to its anisotropic structural design, the FCFe‐G membrane exhibits pronounced directional differences in thermal conductivity. Its in‐plane thermal conductivity reaches 11.46 W·m^−1^·K^−1^, whereas the through‐plane conductivity is only 0.45 W·m^−1^·K^−1^, yielding an anisotropy ratio of 25.47. Additionally, the membrane integrates excellent flame retardancy with temperature‐responsive behavior, offering promising potential for early fire warning applications. By establishing a continuous CNT conductive network, the FCFe‐G membrane reaches steady‐state heating at 156.9 °C under a low driving voltage of 4 V, and 159.7 °C under light power density of 400 mW·cm^−2^, demonstrating applicability in medical hyperthermia, deicing, and antimicrobial fields. This work overcomes the longstanding technical bottleneck of “high reflection–low absorption” in traditional shielding membranes through multigradient structural engineering, laying a theoretical foundation for next‐generation “structure–function–integrated” EMI shielding materials. With its multidimensional integration of mechanical robustness, environmental resilience, and intelligent thermal responsiveness, the FCFe‐G membrane holds strong potential in spacecraft EMI compatibility, flexible electronics packaging, and smart wearable devices, thereby advancing the development of EMI shielding materials toward sustainability, intelligence, and multifunctionality.

## 4 Experimental Section

### Materials and Chemicals

PTFE powder (METABLEN A3800) was provided by Mitsubishi Chemical Corporation, Japan. Single‐walled carbon nanotubes (SWCNTs, outer diameter 1.2–2.0 nm) were supplied by OCSiAl, Russia. Their high conductivity forms the basis for constructing the conductive network. Fe_3_O_4_ (200 nm) was purchased from Macklin Biochemical Technology Co., Ltd., Shanghai. Polylactic acid (PLA, Ingeo4032D), used as a lubricant during the in situ fibrillation process, was provided by NatureWorks, USA. Staphylococcus aureus, used in photothermal antibacterial experiments, was obtained from Luwei Biotechnology Co., Ltd., Shanghai. The chemicals used in the experiments included hydrochloric acid (HCl, 37 wt.%), hydrogen peroxide (H_2_O_2_, 30 wt.%), PBS buffer solution, sodium hydroxide (NaOH, pellets), n‐butanol, and dichloromethane (DCM, chromatographic grade), were purchased from Aladdin Biochemical Technology Co., Ltd., Shanghai.

### Preparation of FCFe Single‐Layer Membranes

Prior to processing, PTFE powder, PLA pellets, CNT, and Fe_3_O_4_ nanoparticles were vacuum‐dried at 80 °C for 12 h to remove residual moisture. To prepare an FCFe membrane containing 10 wt.% CNT and 40 wt.% Fe_3_O_4_, 9 g of PLA was dissolved in 100 mL of DCM under magnetic stirring. Then, 0.2 g of CNT was added, and the mixture was ultrasonicated for 1 h to ensure uniform dispersion. Separately, 9 g PLA and 0.8 g Fe_3_O_4_ were dispersed in 100 mL DCM using the same procedure. The two solutions were combined and heated in a 70 °C water bath for 4 h with continuous mechanical stirring and ultrasonic treatment to obtain a homogeneous CNT/Fe_3_O_4_/PLA/DCM suspension. This suspension was dried in a vacuum oven at 100 °C for 24 h to completely evaporate the DCM. The remaining solids were ground into small granules. Next, 1 g of PTFE powder and 10 g of the CNT/Fe_3_O_4_/PLA composite granules were fed into a twin‐screw extruder and melt‐blended at 190 °C for 10 min. The extrudate was then hot‐pressed into thin films under 10 MPa pressure at 190 °C. The PLA template was removed by Soxhlet extraction in DCM at 65 °C for 20 h, resulting in porous FCFe membranes. To eliminate residual stress introduced during processing, the FCFe membranes were wrapped in 100‐mesh copper and thermally annealed at 320 °C.

By varying the CNT content, FCFe membranes with different CNT loadings were obtained and designated as FCFe‐10 (10 wt.% CNT), FCFe‐20 (20 wt.% CNT), FCFe‐30 (30 wt.% CNT), and FCFe‐40 (30 wt.% CNT). Non‐magnetic control films were also fabricated and labeled as FC‐10, FC‐20, FC‐30, and FC‐40, respectively.

### Preparation of FCFe‐G Multigradient Membranes

The FCFe‐G gradient membranes were fabricated using a layer‐by‐layer assembly process. PTFE/CNT/Fe_3_O_4_/PLA precursor membranes with CNT contents of 10%, 20%, 30%, and 40% were sequentially stacked and hot‐pressed at 190 °C under 15 MPa to form a gradient structure. Subsequently, PLA was removed to yield the FCFe‐G gradient membrane, which was then annealed under a copper mesh to relieve residual stress. To investigate the influence of the multigradient structure on microwave absorption performance, three control groups were designed: (1) a gradient membrane containing only CNT, labeled as FC‐G; (2) a homogeneous membrane containing only CNT, labeled as FC‐H; and (3) a homogeneous membrane containing both CNT and Fe_3_O_4_, labeled as FCFe‐H.

### Characterization and Measurements

The morphological features of FCFe and FCFe‐G membranes, including both surface and cross‐sectional structures, were examined using a field emission scanning electron microscope (FESEM, Gemini 500, Zeiss, Germany). Structural characterization was carried out through wide‐angle X‐ray diffraction (WAXD) using a diffractometer (DMAX‐2500 PC, Rigaku, Japan) over a scanning range of 5–70° at a rate of 5° min^−1^. Following the ASTM D792 standard, density measurements were performed using an analytical balance (AB204, Mettler Toledo, Switzerland). The membrane porosity (*ϵ*) was determined via n‐butanol uptake, and calculated according to Equation (1):^[^ ^31^ ^]^

(1) $$\varepsilon =\frac{\left(m_{1}-m_{2}\right)/\rho_{1}}{\left(m_{1}-m_{2}\right)/\rho_{2}+m_{1}/\rho_{2}}\;$$

where *m*_1_ and *m*_2_ are the weights of the wetted and dry samples, respectively; *ρ*_1_ is the density of n‐butanol (0.8097 g·mL^−1^), and *ρ*_2_ is the composite's average density. The pore structure was further characterized using a capillary flow porometer (CFP‐1500AE, PMI, USA) to determine the average pore size. The water vapor transmission rate (WVTR) was measured using the following equation:^[^ ^31^ ^]^

(2) $$\mathrm{WVTR}\;=\frac{24\times \Delta m}{A\times T}\;$$

where, *Δm* represents the mass loss due to water evaporation, *A* is the effective exposed surface area, and *T* denotes the duration of the test. To evaluate mechanical properties, rectangular specimens (50 mm × 10 mm) were subjected to uniaxial tensile testing at a loading rate of 1 mm min^−1^ using a universal testing machine (HDW‐2000, Hengxu, China). The magnetic behavior of the samples was characterized through hysteresis loop measurements using a Vibrating Sample Magnetometer (VSM 7404, Lake Shore, USA). Dynamic mechanical analysis (DMA, Discovery DMA‐850, TA Instruments, USA) was conducted to assess thermomechanical performance. Specimens (25 mm × 5 mm) were tested in tension from −150 to 300 °C, at a heating rate of 5 °C min^−1^ and a constant frequency of 1 Hz. Surface wettability was studied using a contact angle goniometer (JC2000D, Powereach, China) by placing 3 µL deionized water droplets on the film surface and capturing the dynamic wetting process. Electrical conductivity was evaluated using a four‐point probe system (ST2242, Suzhou Jingge, China).

EMI SE in the X‐band range was measured using a vector network analyzer (N5247A, Agilent, USA). The membranes were positioned within a standard waveguide (22.86 mm × 10.16 mm), secured between two 0.3 mm‐thick PTFE plates due to their flexible nature. The SE values and associated power attenuation parameters were derived from the measured S‐parameters, according to established methods:^[^ ^32^ ^]^

(3) $$SE_{R}=-10\log\left(1-\left|{S_{11}}^{2}\right|\right)$$

(4) $$SE_{A}=-10\log\left(\left|{S_{21}}^{2}\right|/\left(1-\left|{S_{11}}^{2}\right|\right)\right)$$

(5) $$SE_{T}=SE_{R}+SE_{A}+SE_{M}$$

(6) $$R=\left|{S_{11}}^{2}\right|\;$$

(7) $$T=\left|{S_{21}}^{2}\right|\;$$

(8) A=1−R−T

The scattering parameters *S*_11_, *S*_12_ and *S*_21_ were used to calculate the EMI SE. Here, *SE_T_* ​ denotes the total EMI SE, *SE_R_* represents the contribution from reflection, and *SE_A_* ​corresponds to absorption losses. The coefficients *R*, *A*, and *T* refer to the reflected, absorbed, and transmitted power fractions, respectively. When *SE*_T_ exceeds 10 dB, the contribution from multiple internal reflections (*SE_M_* ​) is considered negligible. To enable a fair comparison among shielding materials with varying physical dimensions, the SSE, which accounts for both density and thickness, was calculated using the following equation:

(9) $$SSE=\frac{SE_{T}}{\rho \times W}\;$$

where *ρ* is the material density and *W* is the film thickness. The shielding efficiency, defined as the percentage of EMWs effectively blocked, was determined using:

(10) $$\mathrm{Shielding}\;\mathrm{efficiency}\left(\%\right)=100-\left(\frac{1}{10^{\frac{SE_{T}}{10}}}\right)\times 100$$

Thermal diffusivity (***α***) was measured at 25 °C using a laser flash apparatus (LFA 467, Netzsch, Germany). The thermal conductivity (*λ*) was calculated according to the relation:^[^ ^17^ ^]^

(11) $$\lambda =\rho_{A}\;\times C_{p}\times \alpha$$

where *ρ_A_* and *C_p_* are the density and specific heat of the measured samples. For the Joule heating performance evaluation, a programmable digital source meter (2450, Keithley, USA) was employed to apply voltage. For temperature sensing, the temperature coefficient of resistance (TCR) was determined using Equation (12), while the thermal index (B) was calculated based on Equation (13):^[^ ^36^ ^]^

(12) $$\mathrm{TCR}\;=\frac{1}{R_{0}}\;\times \frac{\Delta R\;}{\Delta T}$$

(13) $$B\;=\frac{\ln\left(R_{0}+\Delta R\right)-\ln R_{0}}{\frac{1}{T_{0}+\Delta T}-\frac{1}{T_{0}}}\;$$

where Δ*R* is the change value of resistors, Δ*T* is the change value of temperature. The slope of the steady‐state temperature versus input power density revealed the heat performance (H_p_) of the composite heater:^[^ ^63^ ^]^

(14) $$H_{p}=\frac{dT\;}{dP}\;$$

where d*T* is the temperature change value, and d*P* is the electric heating power change value. The optical properties, including absorption and reflectance in the 200–2500 nm wavelength range, were characterized using a UV–vis–NIR spectrophotometer (Lambda 950, PerkinElmer, USA), and the UPF value was calculated according to Equation (15):^[^ ^36^ ^]^

(15) $$\mathrm{UPF}\;=\frac{\sum_{\lambda =280}^{\lambda =400}E\;\left(\lambda \right)\cdot \varepsilon \left(\lambda \right)\cdot \Delta \left(\lambda \right)}{\sum_{\lambda =280}^{\lambda =400}E\;\left(\lambda \right)\cdot T\left(\lambda \right)\cdot \varepsilon \left(\lambda \right)\cdot \Delta \left(\lambda \right)}\;$$

where *E*(*λ*) denotes the spectral solar irradiance, *ε*(*λ*) represents the relative effectiveness for erythema, Δ(λ) is the interval between measured wavelengths, and *T*(λ) refers to the transmittance corresponding to the specific wavelength λ. Simulated solar irradiation was generated using a xenon arc lamp (CEL‐HXF300‐T3, Zhongjiao, China). Thermal imaging was conducted with an infrared camera (E8xt, FLIR, USA), while surface temperatures were tracked in real time using a digital thermometer (MIK‐R200T, Supmea, China).

## Conflict of Interest

The authors declare no conflict of interest.

## Supporting information



Supporting Information

Supplemental Video 1

Supplemental Video 2

## Data Availability

The data that support the findings of this study are available from the corresponding author upon reasonable request.
