# Supplementary material for: Tailored Electro–Magnetic–Porous Multigradient Nanoarchitectonics for Absorption‐Dominated Electromagnetic Interference Shielding and Adaptive Multifunctionality
Source: Adv Sci (Weinh). 2025 Aug 14;12(42):e11234. doi: 10.1002/advs.202511234 (PMC12622423; doi:10.1002/advs.202511234)
Supplement: Supplementary file 1 — Supporting Information [file ADVS-12-e11234-s001.docx]

# Supporting information

**Tailored Electro–Magnetic–Porous Multigradient Nanoarchitectonics for Absorption-Dominated Electromagnetic Interference Shielding and Adaptive Multifunctionality**

*Runze Shao, Guilong Wang^*^,Wenyu Wang, Jialong Chai, Guoqun Zhao, Guizhen Wang*

R. Shao, G. Wang, J. Chai, G. Zhao

State key Laboratory of Advanced Equipment and Technology for Metal Forming, Shandong University, Jinan, Shandong 250061, China

R. Shao, G. Wang, J. Chai, G. Zhao

Key Laboratory for Liquid-Solid Structural Evolution and Processing of Materials (Ministry of Education), Shandong University, Jinan, Shandong 250061, China

W. Wang

School of Control Science and Engineering, Shandong University, Jinan, Shandong 250061, China

G. Wang

Center for Advanced Studies in Precision Instruments, Hainan University, Haikou, Hainan 570228, China.

*Corresponding author.

E-mail: [guilong@sdu.edu.cn](mailto:guilong@sdu.edu.cn)

**S1. Theoretical Calculation of EMI Shielding Performance**

The Transfer Matrix Method is employed to calculate the complex reflection coefficient (*R*) and transmission coefficient (*T*) of a homogeneous shielding sample. The electric and magnetic fields of a time harmonic (e^j^*^ωt^*) plane wave, propagating perpendicular to the shields, at the incident face should satisfy the continuity conditions. The continuity of the tangential parts of both fields at the incident face of shields generate the boundary conditions:

$\left\{ \begin{aligned} A_{0i}e^{-ik_{0}z_{0}}+B_{0i}e^{ik_{0}z_{0}}=A_{1}e^{-ik_{1}z_{0}}+B_{1}e^{ik_{1}z_{0}} \\ Y_{0}\left( A_{0i}e^{-ik_{0}z_{0}}-B_{0i}e^{ik_{0}z_{0}} \right)=Y_{1}\left( A_{1}e^{-ik_{1}z_{0}}-B_{1}e^{ik_{1}z_{0}} \right) \end{aligned} \right.$ (1)

where A and B are the coefficients of forward-travelling and backward-travelling waves, $k=\sqrt{\mu\varepsilon}$ is the wave number, $Y=\sqrt{\varepsilon/\mu}$ is the admittance of shields, *μ* and *ε* are the complex permeability and permittivity of shields, the subscripts 0 and 1 are variables relating to the air and the shields, respectively. In terms of the nonmagnetic shields, equals to 1. Moreover, the complex permittivity is composed of the real part and imaginary part:

$\varepsilon=\varepsilon^{'}-j\varepsilon"=\varepsilon^{'}(1-j\frac{\sigma}{\omega\varepsilon^{'}})$ (2)

where w is the angular frequency and σ is the conductivity of shields. Here, we set the real part (*ε* ') equal to the with *ε*_0_, leading to a conductivity-caused EMI shielding calculation of the homogenous shields. At the wave emergent face of the shields, the boundary condition gives:

$\left\{ \begin{aligned} A_{1}e^{-ik_{1}z_{1}}+B_{1}e^{ik_{1}z_{1}}=A_{0t}e^{-ik_{0}z_{1}} \\ Y_{1}\left( A_{1}e^{-ik_{1}z_{1}}-B_{1}e^{ik_{1}z_{1}} \right)=Y_{0}A_{0t}e^{-ik_{0}z_{1}} \end{aligned} \right.$ (3)

Therefore, the complex reflection coefficient *R* and transmission coefficient *T* of the shields can be calculated as：

$R=\frac{B_{0r}}{A_{0i}}$ (4)

$T=\frac{A_{0t}}{A_{0i}}$ (5)

Furthermore, the *SE_T_* and *SE_R_* of the shields in dB can be calculated:

${SE}_{T}=10\log\frac{1}{T^{2}}$ (6)

${SE}_{R}=10\log\frac{1}{{1-R}^{2}}$ (7)

**S2. EM Simulation Settings and Parameters**

The EM simulation was conducted using CST STUDIO SUITE 2023. The model dimensions were set to a length of 22.86 mm, a width of 10.16 mm, and a thickness of 140 mm. The surrounding space parameters were defined with lower and upper X and Y distances of 0, and lower and upper Z distances of 80 mm. Boundary conditions were applied with electric field (E_t_=0) constraints at X_min_, X_max_, Y_min_, and Y_max_, while Z_min_ and Z_max_ were set to "open (add space)" conditions. The simulation frequency range was specified from 8.2 GHz to 12.4 GHz. Waveguide ports were configured with Port 1 aligned along the Z-axis with a negative orientation, and Port 2 aligned along the Z-axis with a positive orientation, both spanning the full plane. Field monitors were set at a frequency of 10.3 GHz to capture the electric field, magnetic field, power, and surface current. The frequency domain solver employed a broadband sweep with a general-purpose tetrahedral mesh, and the excitation source was set to Port 1, with all modes enabled.

The film model was centrally positioned within the waveguide cavity, with the FCFe-10 side facing Port 1 and the FCFe-40 side facing Port 2. The thicknesses of FCFe-10, FCFe-20, FCFe-30, and FCFe-40 were 28.3, 24.9, 25.3, and 22.6 µm, respectively. Material parameters for all components were defined using either the raw datasets obtained from different samples or the material parameter library provided within the software.

Due to computational limitations, the model was scaled down while preserving the original aspect ratio and adjusted to an appropriate thickness. As a result, a single FCFe porous material model with dimensions of 19.2 × 9.6 × 4.8 mm³ was constructed. During the modeling process, to ensure uniform density across FCFe porous materials with varying pore sizes, the pores were arranged in a vertically aligned array within a Cartesian coordinate system. This approach guaranteed that, regardless of pore diameter, the remaining solid volume after pore removal remained constant, thereby eliminating the influence of porosity on the material’s EMI shielding performance. Vertically aligned porous models were generated with pore diameters of 1.2, 1.6, 2.4, and 4.8 mm. These models were subsequently imported into CST Microwave Studio for EMI shielding simulations. To isolate the effect of pore size gradient on EMI performance, all porous materials with different pore diameters were assigned identical EM parameters.

**S3. The photothermal antibacterial experiment**

The photothermal antibacterial performance was evaluated using Staphylococcus aureus as the model bacterium. First, a suspension of S. aureus was uniformly spread onto the surface of agar plates. The FCFe-G composite and pure PTFE were then placed at the center of the inoculated plates. The FCFe-G membrane was irradiated with a xenon lamp, raising its surface temperature to 60 °C, which was maintained for 20 min. Subsequently, the plates were incubated at 37 °C for 12 h. After incubation, the bacterial growth and the presence of an inhibition zone around the film were visually examined to assess the antibacterial efficacy.


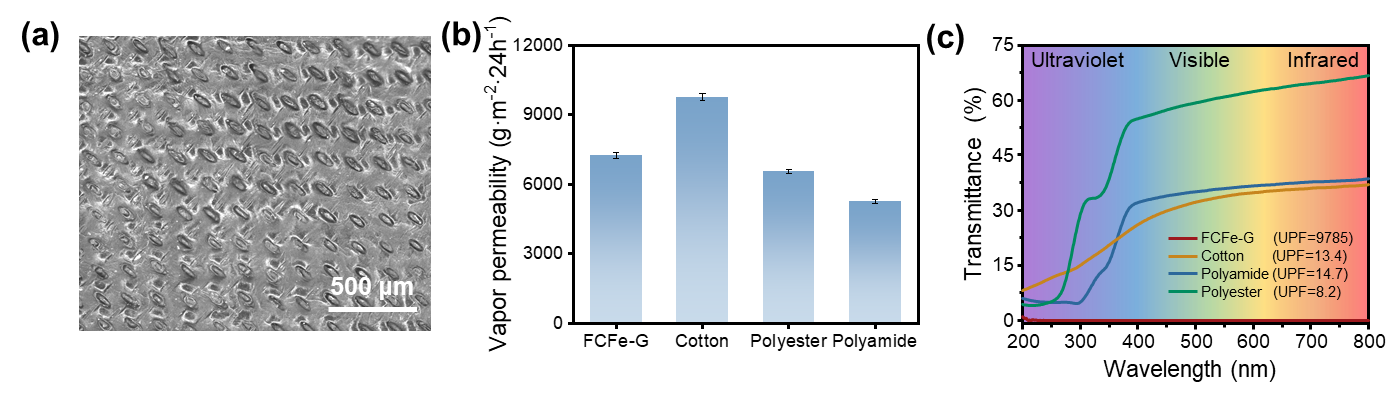


**Figure S1**. (a) Surface texture of the FCFe-G membrane after copper mesh-assisted annealing. (b) Comparison of water vapor transmission rates between the FCFe-G membrane and common textiles. (c) UV–vis–NIR transmittance spectra of the FCFe-G membrane and commercial textiles; the inset shows the corresponding ultraviolet protection factor.


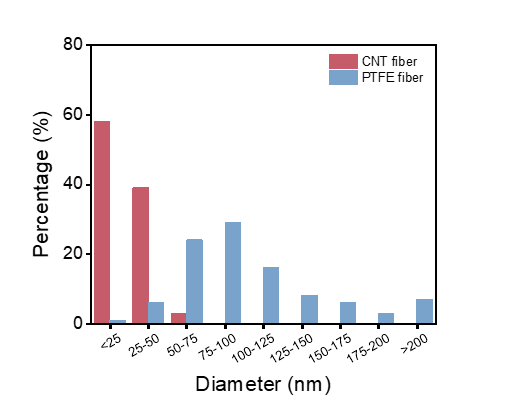


**Figure S2**. Diameter distribution of the CNT fiber and PTFE fiber in the FCFe dual-nanofibrous network.


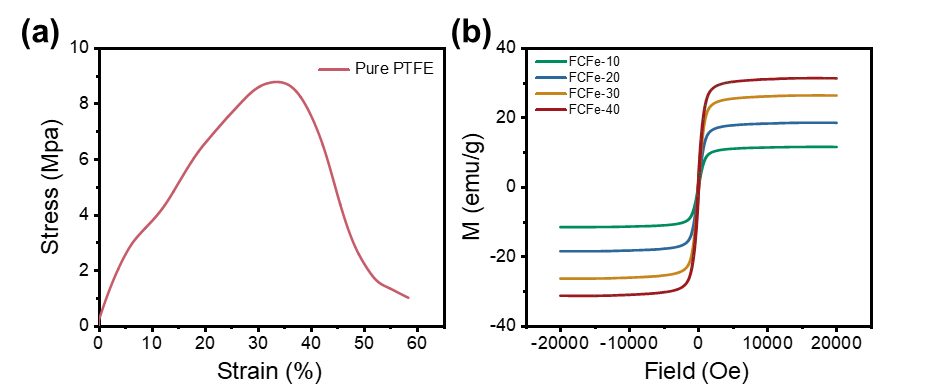


**Figure S3**. (a)Stress-strain curves of pure PTFE film. (b) Hysteresis loops of FCFe membranes.


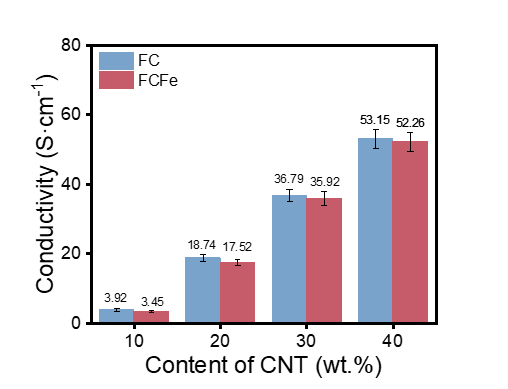


**Figure S4**. The electrical conductivity of FC and FCFe membranes with different CNT contents.


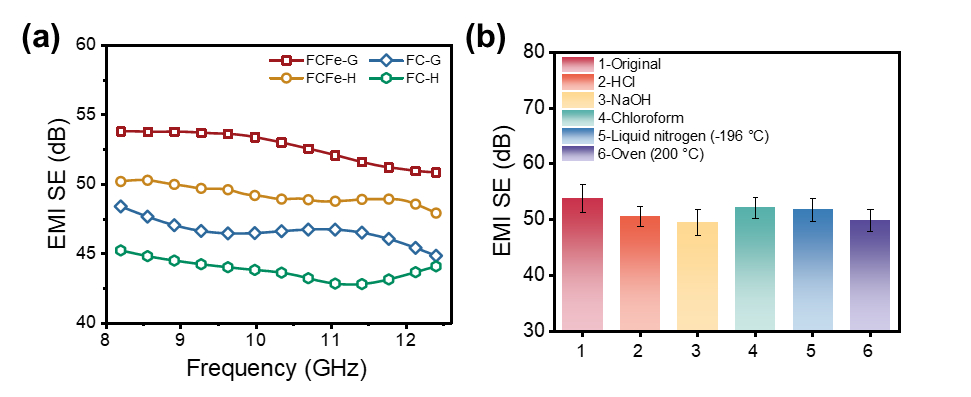


**Figure. S5**. (a) The EMI SE of FCFe-G, FCFe-H, FC-G and FC-H. (b) EMI SE variation of the FCFe-G membrane after the durability tests.


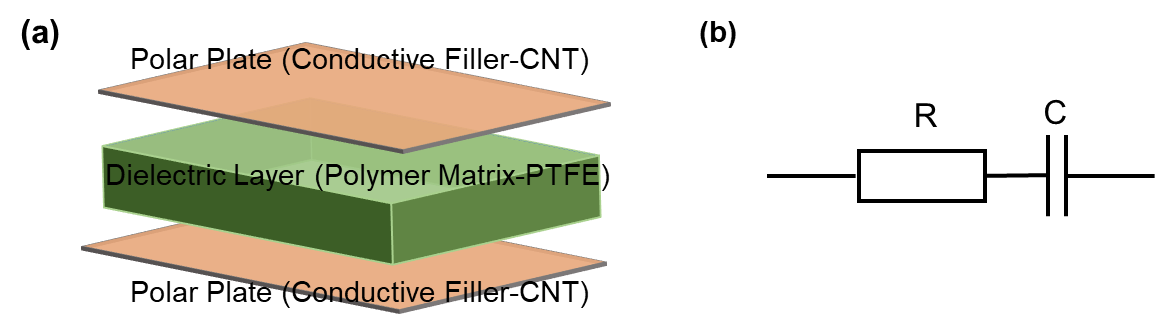


**Figure S6**. (a) The microcapacitor structure model. (b)The R-C model of microcapacitor.


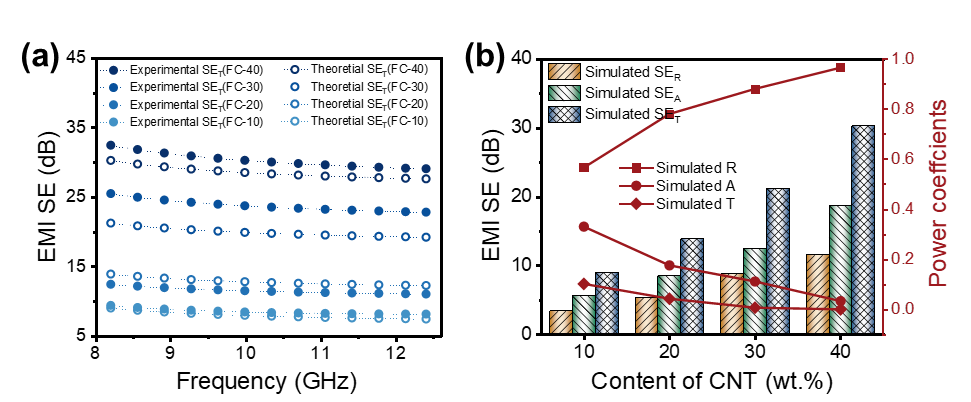


**Figure. S7**. (a) Simulated EMI SE of FC membranes with varying CNT contents. (b) Simulated EMI shielding performance (SE_T_, SE_A_, and SE_R_) and power coefficients (R, T, and A) of FC membranes.


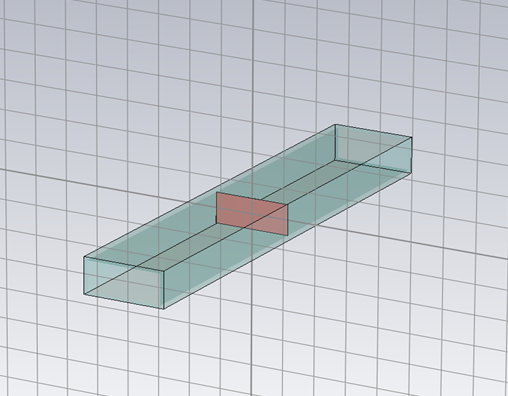


**Figure S8**. Schematic of modeling in CST simulation.


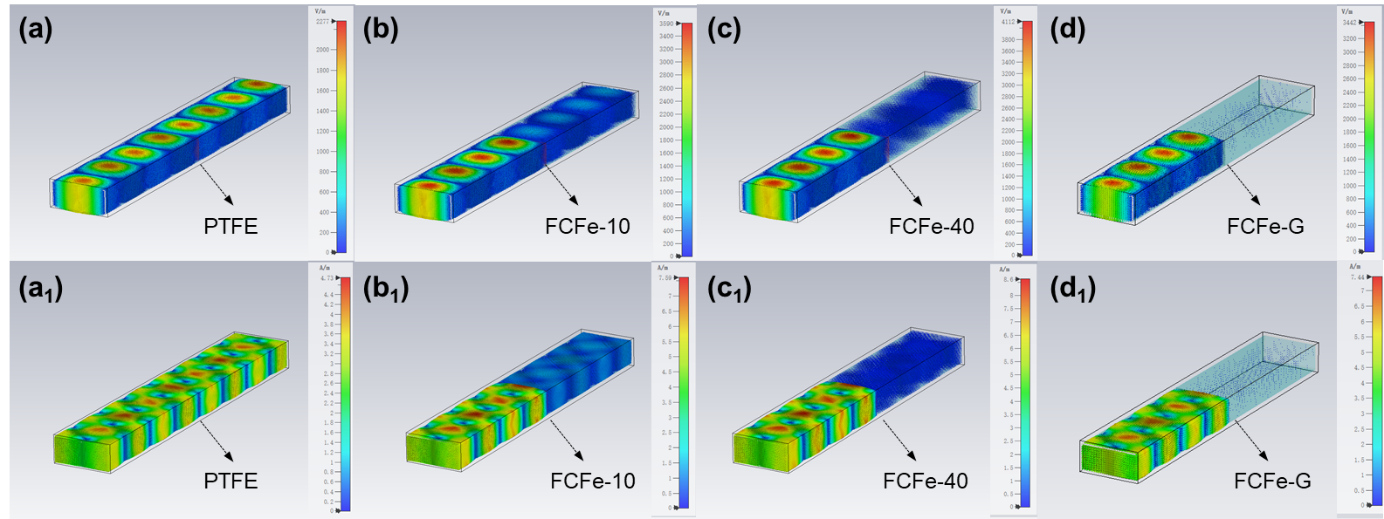


**Figure S9**. The electric fields of (a) PTFE, (b) FCFe-10, (c) FCFe-40m, and (d) FCFe-G in X-band. The magnetic fields of (a_1_) PTFE, (b_1_) FCFe-10, (c_1_) FCFe-40, and (d_1_) FCFe-G in X-band


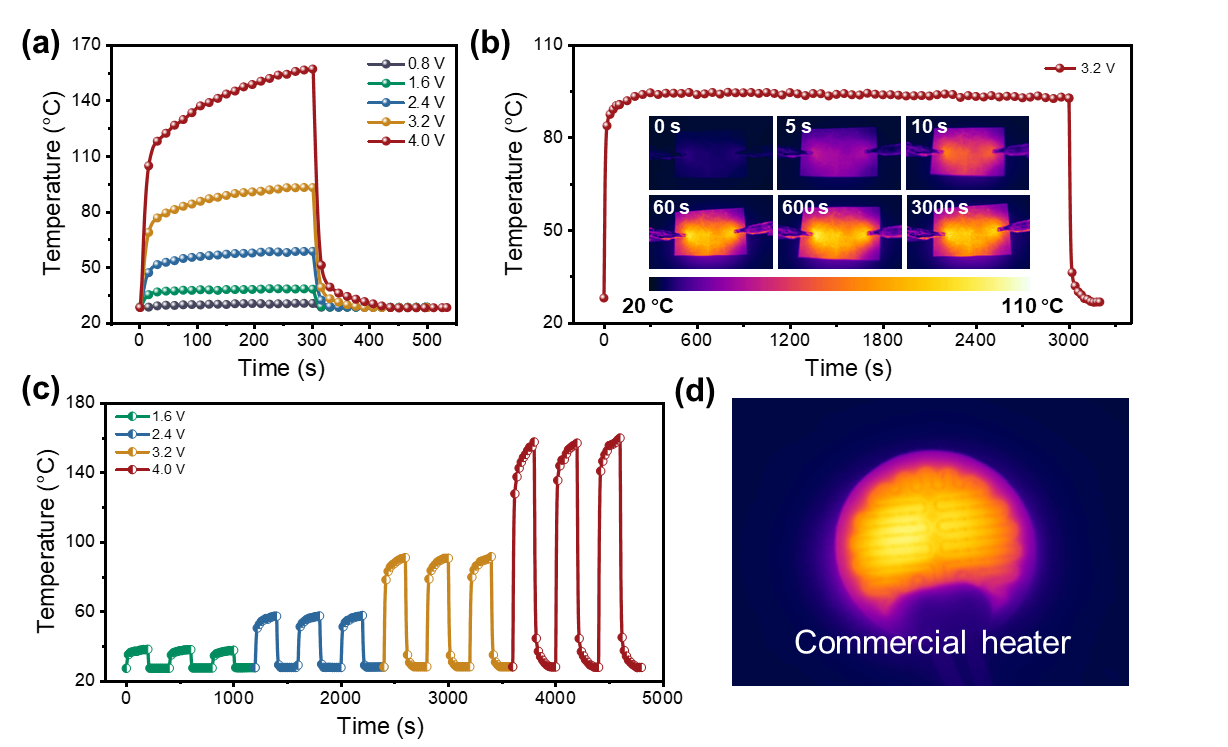


**Figure S10**. (a) The temperature variation curves of the FCFe-G membrane at different voltages. (b) The long-term heating stability tests at a voltage of 3.2 V for FCFe-G. (c) The cyclic stability experiments of the FCFe-G membrane. (d) The infrared image of commercial heater.


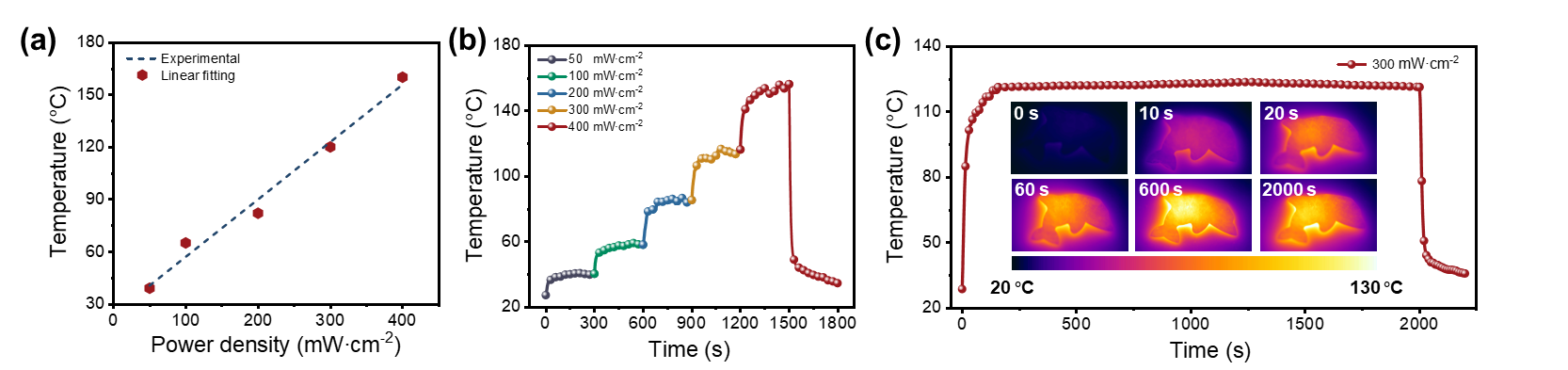


**Figure S11**. (a) Experimental data and fitting linear of saturation temperature versus light power density. (b) Surface temperature-time variation curves of the FCFe-G after changes in optical power density gradients. (c) Long-term photothermal stability testing of the FCFe-G.

**Table S1**. Density of FCFe and FCFe-G membranes

| Sample | Density (g·cm^-3^) |
| --- | --- |
| FCFe-10 | 0.652 |
| FCFe-20 | 0.573 |
| FCFe-30 | 0.533 |
| FCFe-40 | 0.485 |
| FCFe-G | 0.558 |

**Table S2**. Mechanical properties of pure PTFE, FCFe and FCFe-G membranes.

| Sample | Tensile strength  (MPa) | Elongation at break  (%) | Young’s modulus (MPa) |
| --- | --- | --- | --- |
| PTFE | 8.78 | 58.27 | 12.16 |
| FCFe-10 | 11.52 | 43.41 | 63.13 |
| FCFe-20 | 15.92 | 38.55 | 212.76 |
| FCFe-30 | 27.64 | 27.75 | 372.16 |
| FCFe-40 | 17.79 | 22.61 | 398.46 |
| FCFe-G | 26.12 | 30.52 | 158.49 |

**Table S3**. EMI shielding performance of various shielding materials.

| Materials | Thickness (mm) | EMI SE  (dB) | SSE  (dB·cm^2^·g^−1^) | Ref. |
| --- | --- | --- | --- | --- |
| FCFe-10 | 0.047 | 10.20 | 3325.589 | This work |
| FCFe-20 | 0.051 | 15.64 | 5345.698 | This work |
| FCFe-30 | 0.048 | 25.37 | 9903.904 | This work |
| FCFe-40 | 0.053 | 33.09 | 12864.49 | This work |
| **FCFe-G** | **0.101** | **53.79** | **9539.52** | **This work** |
| MXene/CNF | 0.047-0.167 | 23.5-25.8 | 1326-2647 | [1] |
| MXene/MMT | 0.033-0.045 | 28-46 | 3254-6336 | [2] |
| MXene/PVA | 0.1-0.3 | 26-28 | 3867-4770 | [3] |
| MXene/TONCF | 0.047 | 32.7 | 4761 | [4] |
| Graphene/WPU | 2 | 32 | 153 | [5] |
| Graphene/PMMA | 3.4 | 30 | 74 | [6] |
| RGO-γ-Fe_2_O_3_ | 0.36 | 20.3 | 416.7 | [7] |
| RGO/Fe_3_O_4_ | 1.8 | 13 | 49.5 | [8] |
| AgNWs/PI | 5 | 35 | 2416 | [9] |
| AgNWs/cellulose | 0.16 | 48.6 | 5585 | [10] |
| CNT/AgNWs/cellulose | 0.16 | 23.8 | 2916.7 | [11] |
| MWCNT/PLLA | 2.5 | 23 | 306.7 | [12] |
| CNTs/PC | 1.85 | 25 | 112.6 | [13] |
| CNTs/PP | 1 | 35 | 372 | [14] |
| MWCNT/CNF | 0.15 | 46.4 | 4017.3 | [15] |
| MWCNT/cellulose aerogel | 2.5 | 20-35 | 1700-3776 | [16] |
| CNT/PMMA | 4.5 | 30 | 49 | [17] |
| CNT/WPU | 0.32 | 35 | 779 | [18] |
| CNT/WPU foam | 2.3 | 50.5 | 1743 | [19] |

## References

[1] W.-T. Cao, F.-F. Chen, Y.-J. Zhu, Y.-G. Zhang, Y.-Y. Jiang, M.-G. Ma, F. Chen, *ACS Nano* **2018**, 12, 4583.

[2] L. Li, Y. Cao, X. Liu, J. Wang, Y. Yang, W. Wang, *ACS Applied Materials & Interfaces* **2020**, 12, 27350.

[3] H. Xu, X. Yin, X. Li, M. Li, S. Liang, L. Zhang, L. Cheng, *ACS Appl. Mater. Interfaces* **2019**, 11, 10198.

[4] Z. Zhan, Q. Song, Z. Zhou, C. Lu, *J. Mater. Chem. C* **2019**, 7, 9820.

[5] S.-T. Hsiao, C.-C. M. Ma, H.-W. Tien, W.-H. Liao, Y.-S. Wang, S.-M. Li, Y.-C. Huang, *Carbon* **2013**, 60, 57.

[6] H.-B. Zhang, W.-G. Zheng, Q. Yan, Z.-G. Jiang, Z.-Z. Yu, *Carbon* **2012**, 50, 5117.

[7] B. Yuan, C. Bao, X. Qian, L. Song, Q. Tai, K. M. Liew, Y. Hu, *Carbon* **2014**, 75, 178.

[8] K. Yao, J. Gong, N. Tian, Y. Lin, X. Wen, Z. Jiang, H. Na, T. Tang, *RSC Adv.* **2015**, 5, 31910.

[9] J. Ma, K. Wang, M. Zhan, *RSC Adv.* **2015**, 5, 65283.

[10] T.-W. Lee, S.-E. Lee, Y. G. Jeong, *ACS Appl. Mater. Interfaces* **2016**, 8, 13123.

[11] H. Y. Choi, T.-W. Lee, S.-E. Lee, J. Lim, Y. G. Jeong, *Compos. Sci. Technol.* **2017**, 150, 45.

[12] T. Kuang, L. Chang, F. Chen, Y. Sheng, D. Fu, X. Peng, *Carbon* **2016**, 105, 305.

[13] M. Arjmand, M. Mahmoodi, G. A. Gelves, S. Park, U. Sundararaj, *Carbon* **2011**, 49, 3430.

[14] M. H. Al-Saleh, U. Sundararaj, *Carbon* **2009**, 47, 1738.

[15] H. Zhang, X. Sun, Z. Heng, Y. Chen, H. Zou, M. Liang, *Ind. Eng. Chem. Res.* **2018**, 57, 17152.

[16] L.-Q. Zhang, S.-G. Yang, L. Li, B. Yang, H.-D. Huang, D.-X. Yan, G.-J. Zhong, L. Xu, Z.-M. Li, *ACS Appl. Mater. Interfaces* **2018**, 10, 40156.

[17] N. C. Das, Y. Liu, K. Yang, W. Peng, S. Maiti, H. Wang, *Polym. Eng. Sci.* **2009**, 49, 1627.

[18] Z. Zeng, M. Chen, H. Jin, W. Li, X. Xue, L. Zhou, Y. Pei, H. Zhang, Z. Zhang, *Carbon* **2016**, 96, 768.

[19] Z. Zeng, H. Jin, M. Chen, W. Li, L. Zhou, Z. Zhang, *Adv. Funct. Mater.* **2015**, 26, 303.

[1] W.-T. Cao, F.-F. Chen, Y.-J. Zhu, Y.-G. Zhang, Y.-Y. Jiang, M.-G. Ma, F. Chen, "Binary Strengthening and Toughening of MXene/Cellulose Nanofiber Composite Paper with Nacre-Inspired Structure and Superior Electromagnetic Interference Shielding Properties," *ACS Nano* **2018**, *12* (5), 4583.

https://doi.org/10.1021/acsnano.8b00997

[2] L. Li, Y. Cao, X. Liu, J. Wang, Y. Yang, W. Wang, "Multifunctional MXene-Based Fireproof Electromagnetic Shielding Films with Exceptional Anisotropic Heat Dissipation Capability and Joule Heating Performance," *ACS Appl. Mater. Interfaces* **2020**, *12* (24), 27350.

https://doi.org/10.1021/acsami.0c05692

[3] H. Xu, X. Yin, X. Li, M. Li, S. Liang, L. Zhang, L. Cheng, "Lightweight Ti_2_CT_x_ MXene/Poly(vinyl alcohol) Composite Foams for Electromagnetic Wave Shielding with Absorption-Dominated Feature," *ACS Appl. Mater. Interfaces* **2019**, *11* (10), 10198.

https://doi.org/10.1021/acsami.8b21671

[4] Z. Zhan, Q. Song, Z. Zhou, C. Lu, "Ultrastrong and Conductive MXene/Cellulose Nanofiber Films Enhanced by Hierarchical Nano-Architecture and Interfacial Interaction for Flexible Electromagnetic Interference Shielding," *J. Mater. Chem. C* **2019**, *7* (32), 9820.

https://doi.org/10.1039/c9tc03309b

[5] S.-T. Hsiao, C.-C. M. Ma, H.-W. Tien, W.-H. Liao, Y.-S. Wang, S.-M. Li, Y.-C. Huang, "Using a Non-covalent Modification to Prepare a High Electromagnetic Interference Shielding Performance Graphene Nanosheet/Water-Borne Polyurethane Composite," *Carbon* **2013**, *60*, 57.

https://doi.org/10.1016/j.carbon.2013.03.056

[6] H.-B. Zhang, W.-G. Zheng, Q. Yan, Z.-G. Jiang, Z.-Z. Yu, "The Effect of Surface Chemistry of Graphene on Rheological and Electrical Properties of Polymethylmethacrylate Composites," *Carbon* **2012**, *50* (14), 5117.

https://doi.org/10.1016/j.carbon.2012.06.052

[7] B. Yuan, C. Bao, X. Qian, L. Song, Q. Tai, K. M. Liew, Y. Hu, "Design of Artificial Nacre-Like Hybrid Films as Shielding to Mitigate Electromagnetic Pollution," *Carbon* **2014**, *75*, 178.

https://doi.org/10.1016/j.carbon.2014.03.051

[8] K. Yao, J. Gong, N. Tian, Y. Lin, X. Wen, Z. Jiang, H. Na, T. Tang, "Flammability Properties and Electromagnetic Interference Shielding of PVC/Graphene Composites Containing Fe_3_O_4_ nanoparticles," *RSC Adv.* **2015**, *5* (40), 31910.

https://doi.org/10.1039/c5ra01046b

[9] J. Ma, K. Wang, M. Zhan, "A Comparative Study of Structure and Electromagnetic Interference Shielding Performance for Silver Nanostructure Hybrid Polyimide Foams," *RSC Adv.* **2015**, *5* (80), 65283.

https://doi.org/10.1039/c5ra09507g

[10] T.-W. Lee, S.-E. Lee, Y. G. Jeong, "Highly Effective Electromagnetic Interference Shielding Materials based on Silver Nanowire/Cellulose Papers," *ACS Appl. Mater. Interfaces* **2016**, *8* (20), 13123.

https://doi.org/10.1021/acsami.6b02218

[11] H. Y. Choi, T.-W. Lee, S.-E. Lee, J. Lim, Y. G. Jeong, "Silver Nanowire/Carbon Nanotube/Cellulose Hybrid Papers for Electrically Conductive and Electromagnetic Interference Shielding Elements," *Compos. Sci. Technol.* **2017**, *150*, 45.

https://doi.org/10.1016/j.compscitech.2017.07.008

[12] T. Kuang, L. Chang, F. Chen, Y. Sheng, D. Fu, X. Peng, "Facile Preparation of Lightweight High-Strength Biodegradable Polymer/Multi-Walled Carbon Nanotubes Nanocomposite Foams for Electromagnetic Interference Shielding," *Carbon* **2016**, *105*, 305.

https://doi.org/10.1016/j.carbon.2016.04.052

[13] M. Arjmand, M. Mahmoodi, G. A. Gelves, S. Park, U. Sundararaj, "Electrical and Electromagnetic Interference Shielding Properties of Flow-Induced Oriented Carbon Nanotubes in Polycarbonate," *Carbon* **2011**, *49* (11), 3430.

https://doi.org/10.1016/j.carbon.2011.04.039

[14] M. H. Al-Saleh, U. Sundararaj, "Electromagnetic Interference Shielding Mechanisms of CNT/Polymer Composites," *Carbon* **2009**, *47* (7), 1738.

https://doi.org/10.1016/j.carbon.2009.02.030

[15] H. Zhang, X. Sun, Z. Heng, Y. Chen, H. Zou, M. Liang, "Robust and Flexible Cellulose Nanofiber/Multiwalled Carbon Nanotube Film for High-Performance Electromagnetic Interference Shielding," *Ind. Eng. Chem. Res.* **2018**, *57* (50), 17152.

https://doi.org/10.1021/acs.iecr.8b04573

[16] L.-Q. Zhang, S.-G. Yang, L. Li, B. Yang, H.-D. Huang, D.-X. Yan, G.-J. Zhong, L. Xu, Z.-M. Li, "Ultralight Cellulose Porous Composites with Manipulated Porous Structure and Carbon Nanotube Distribution for Promising Electromagnetic Interference Shielding," *ACS Appl. Mater. Interfaces* **2018**, *10* (46), 40156.

https://doi.org/10.1021/acsami.8b14738

[17] N. C. Das, Y. Liu, K. Yang, W. Peng, S. Maiti, H. Wang, "Single‐Walled Carbon Nanotube/Poly(Methyl Methacrylate) Composites for Electromagnetic Interference Shielding," *Polym. Eng. Sci.* **2009**, *49* (8), 1627.

https://doi.org/10.1002/pen.21384

[18] Z. Zeng, M. Chen, H. Jin, W. Li, X. Xue, L. Zhou, Y. Pei, H. Zhang, Z. Zhang, "Thin and Flexible Multi-Walled Carbon Nanotube/Waterborne Polyurethane Composites with High-Performance Electromagnetic Interference Shielding," *Carbon* **2016**, *96*, 768.

https://doi.org/10.1016/j.carbon.2015.10.004

[19] Z. Zeng, H. Jin, M. Chen, W. Li, L. Zhou, Z. Zhang, "Lightweight and Anisotropic Porous MWCNT/WPU Composites for Ultrahigh Performance Electromagnetic Interference Shielding," *Adv. Funct. Mater.* **2015**, *26* (2), 303.

https://doi.org/10.1002/adfm.201503579
